# New species of *Ipomoea* (Convolvulaceae) from South America

**DOI:** 10.3897/phytokeys.88.12891

**Published:** 2017-10-11

**Authors:** John R.I. Wood, Pablo Muñoz-Rodríguez, Rosa Degen, Robert W. Scotland

**Affiliations:** 1 Department of Plant Sciences, University of Oxford, South Parks Road, Oxford, OX1 3RB, UK; 2 Honorary Research Associate, Royal Botanic Gardens, Kew, Richmond, Surrey, TW9 3AB, UK; 3 Facultad de Ciencias Químicas, Departamento de Botánica, Universidad Nacional de Asunción, Herbario FCQ, Casilla de Correo Campus UNA, 11001-3291, Paraguay

**Keywords:** Brazil, Caatinga, Cerrado, Chapada dos Veadeiros, endemic, inselbergs, Paraguay, Peru, taxonomy

## Abstract

The importance of discovering, describing and cataloguing poorly known species in herbarium collections is discussed. It is a spur to efforts at rediscovery and consequent conservation efforts. The problems faced in describing species from limited material are discussed and our methods and criteria in making a decision are described. Prospects for future novelties are briefly assessed. Fifteen new species are described and illustrated with line drawings and distribution maps: *Ipomoea attenuata* J.R.I. Wood & Scotland, *I. cuscoensis* J.R.I. Wood & P. Muñoz, *I. dasycarpa* J.R.I. Wood & Scotland, *I. dolichopoda* J.R.I. Wood & R. Degen, *I. ensiformis* J.R.I.Wood & Scotland, *I. fasciculata* J.R.I. Wood & Scotland, *I. graminifolia* J.R.I. Wood & Scotland, *I. kraholandica* J.R.I. Wood & Scotland, *I. longirostra* J.R.I. Wood & Scotland, *I. revoluta* J.R.I. Wood & Scotland, *I. scopulina* J.R.I. Wood &. Scotland, *I. uninervis* J.R.I. Wood & Scotland, *I. veadeirosii* J.R.I. Wood & Scotland, *I. velutinifolia* J.R.I. Wood & Scotland, *I. walteri* J.R.I. Wood & Scotland. All species are narrow endemics except *I. velutinifolia* which is found in Brazil and Peru; of the others, 12 are found in Brazil and one each in Paraguay and Peru.

## Introduction

*Ipomoea* is a large genus in the Americas, where it is represented by around 430 species (Wood and Scotland 2017c). It is almost entirely restricted to the tropical and subtropical regions, and our studies show that the greatest diversity lies between latitudes 15° and 30° both south and north of the equator (Wood and Scotland 2017c).

Several recent papers have highlighted the number of unidentified or wrongly identified specimens lying in the cupboards of the world’s herbaria (Bebber et al. 2010; Goodwin et al. 2015). Attention has been drawn to the importance of revising these, often old, collections, which may contain significant numbers of unrecognised new species that may subsequently be rediscovered as a result of focussed field work (Cavallin et al. 2016). Only by completing this task are we likely to get significantly closer to completing the inventory of the world’s flowering plants and reaching a comprehensive understanding of the conservation threats faced by the world’s flora.

As part of our monographic studies of *Ipomoea* at Oxford, we regularly come across unidentified specimens in material sent for determination or examined during visits to different herbaria. The majority of these unnamed specimens are readily identified but there usually remains a residue of problematic collections. Some of these are essentially unidentifiable, usually because they are sterile or lacking important diagnostic characters. In other cases, the specimen represents an atypical form from a complex of species whose delimitation is problematic. However, it is quite common to find specimens of what appear to be undescribed new species.

In an ideal world, the researcher would set out to find living material of these putative new species. These efforts are sometimes successful, as in the case of the plant first collected by Jim Solomon in 1985, then recollected, sequenced and described as *I. lactifera* J.R.I. Wood & Scotland after rediscovery in 2014 (Wood et al 2015). More common is the first author’s experience of a frustrating day trying to refind *I. gypsophila* J.R.I. Wood & Scotland in the field near Tarija, in Bolivia, after “discovering” it in the Lillo herbarium (LIL) in Tucuman, Argentina (Wood et al. 2015: 61). There are many factors which make efforts at rediscovery by a single researcher impractical if not impossible, including time, budgetary considerations, the complexities of getting collecting permits, the imprecision, inaccessibility or destruction of the original collection locality and so on. However, once a species is formally described and recognised, there exists a specific biological entity, which can constitute the target of efforts at rediscovery. The experience of the first author in different regions of the world from the Himalayas and Arabia to South America is that many long-lost species can be refound after informed and focussed search, but the knowledge that the species exists is a prerequisite for success.

It should also be stressed that unless a species is formally described it does not exist as an entity to be considered for conservation. A nameless plant cannot be catalogued or red-listed. The threats to its existence cannot be assessed. It adds nothing to the conservation value of a particular habitat, locality or protected area. In contrast, the description in this paper of four new species endemic to the Chapada dos Veadeiros National Park augments the biological importance of this national park as a centre of endemism and a priority for conservation. Similarly, the description of two new species from isolated inselbergs in eastern Brazil draws attention to the importance of this type of habitat as a reservoir of rare endemic species.

If a putative new species cannot be immediately refound, a decision has to be made as to whether or not to go ahead with a description using only the herbarium specimen(s). The decision is, of course, made easier if more than a single specimen is known, especially if the specimens come from different localities. More collections provide greater certainty in diagnosing a new taxon. However, in many cases a single collection is all there is. A decision then has to be made based on whatever evidence is available. Sometimes analysis of DNA sequences can provide support for the description of a new species, if fresh or recently collected material is available as in the case of *I. kraholandica* described below. In other cases, a decision has to be made based entirely on morphological evidence, a decision that depends ultimately on the authors’ knowledge of the group and the characters that are significant in species delimitation, characters that are often not constant throughout the genus but which may be specific to a particular clade, group or subgroup. To some extent, our comprehensive monographic studies and knowledge of species-level variation gives us confidence that our description of new species based on limited material is sound. However, there is a degree of subjectivity involved and only the perspective of time can show how reliable the taxonomist’s judgement was. Amongst major neotropical taxonomists working on *Ipomoea*, the data would suggest that Choisy and Meisner’s decisions were unreliable, House’s were better, whereas O’Donell’s were usually very sound (Wood 2017). Our own judgements will no doubt be evaluated over time based on how well they have accounted for the variation found in *Ipomoea*.

This paper describes twelve new species of *Ipomoea* from Brazil, one from Brazil and Peru and one each from Paraguay and Peru, all based on herbarium specimens, mostly found in Brazilian herbaria. There is little doubt that other species will be found as the American flora, the Brazilian in particular, is explored more intensively. The Checklist of the Brazilian Flora (Forzza et al. 2010) listed 118 species, but since its publication many further species have been added (Vasconcelos et al. 2016, Wood et al. 2015, 2016, 2017d, Wood and Scotland 2017a, 2017b) so that the total had reached about 150 by the middle of 2017. Similarly, 44 species of *Ipomoea* were listed for Paraguay in the Catálogo del Cono Sur (Austin 2008) but a recent checklist has raised this number to 61 (Wood et al. 2017e). Most of these species come from the Cerrado and Caatinga biomes, the rarity of new species in Amazonia being striking (Maps 1, 2). Here we add a further fifteen species to the list of novelties, mostly from these two biomes. This is far from being the total number of Brazilian plants which cannot be assigned to a recognised species so it is likely that further new species will be described in the future although at a diminishing rate.

## Materials and methods

The species described in this paper are only known to the authors from herbarium specimens deposited at ARIZ, CEN, CUZ, E, FTG, K, MG, MICH, MO, MSM, NY, RB, SPF and UB. In every case we have examined herbarium specimens to prepare the descriptions but have sometimes cited duplicates based on digital images provided by various herbaria. Specimens have been examined using a binocular microscope but limited dissection has been carried because of the state of the material. When possible, we obtained DNA sequences from a few of these new species, specifically using standard Sanger sequencing to sequence the nuclear ribosomal Internal Transcribed Spacer (*ITS*), which had proved useful in *Ipomoea* before (Miller et al. 1999; Manos et al. 2001). This has helped us to confirm the relationships of some species. All cited specimens have been seen by the authors unless indicated otherwise. Barcodes have been added where known. Most species in this paper are too poorly known for a realistic conservation assessment following IUCN guidelines (2012) but it is likely that many are Black Star species according to the system elaborated by William Hawthorne (Hawthorne and Marshall 2016), which is in many ways more satisfactory at categorizing rare, poorly-known species such as those described in this present paper.

The descriptions that follow are based on limited material and, in most cases, very sparse field notes. Information about size, habit (and habitat) often has to be inferred. Rootstock, fruit and seeds are often missing and unknown. The range of variation in dimensions may not prove completely correct once a larger number of specimens becomes available. Dissection of flowers is often problematic as the specimen may have only one or two corollas pasted to the sheet and any attempt at dissection may result in the destruction of the flower, given the fragile nature of most *Ipomoea* corollas. Fortunately, diagnostic information is rarely provided by detailed study of the stamens or style; the filaments seem always to be unequal (two long, three short) with included anthers in species with a funnel-shaped corolla; they are nearly always hairy at the base only; the ovary is usually glabrous, 2-locular with 4 ovules and the stigma obscurely 2-lobed and subglobose in form. Exceptions are rare and mostly found in the well-marked clades such as *Quamoclit*, *Batatas* and *Pharbitis*, which are not represented among the species described below.

Table 1 has been provided to show the differences between three of the new species and *I. campestris* Meisn., with which they appear to be related. This table should be treated with caution as two of the species are only known from single collections and so the range of variation is not fully known. In general, we have tried to highlight so-called “conservative” or diagnostic characters in the notes which accompany each species description. Amongst characters which we regard as diagnostic are the shape, indumentum and relative sizes of the sepals, the shape and external indumentum of the corolla, the form of a well-developed inflorescence, the indumentum of the ovary and capsule and the indumentum of the seeds. We have relied on these “conservative” characters as far as possible.

**Table 1.** Differences between *Ipomoea campestris*, *I. attenuata*, *I. dolichopoda* and *I. ensiformis*.

	*I. campestris*	*I. attenuata*	*I. dolichopoda*	*I. ensiformis*
Habit	usually erect, sometimes decumbent	decumbent or climbing	decumbent	procumbent
Indumentum	pubescent	pubescent	pilose with long spreading white hairs	thinly pubescent, glabrescent
Leaf size	4–10 × 0.1–1 cm	4–10 × 0.3–0.7 cm	4–6.5 × 0.8–1.5 cm	2–6 × 0.3–1.6 cm
Inflorescence	simple axillary cymes of 1–2 flowers	elongate, complex cymes, racemose in form	axillary cymes with long secondary peduncles	simple axillary cymes of 1(–2) flowers
Peduncles	0.2–1.5 cm, relatively stout	2–7 cm	0.3–12 cm	1.4–3.2 cm
Bracteoles	Linear, 1–2 mm, caducous	Linear-lanceolate, 4–7 mm, persistent	filiform, 9–12 mm, tardily caducous	ovate, 3 mm, tardily caducous
Pedicels	3–9 mm	3–5 mm	8–15 mm	5–6 mm
Sepal length	8–11 mm	11–14 mm	10–14 mm	6–8 mm
Sepal shape	oblong-ovate, apex acute, base rounded	ovate, apex acuminate to attenuate, base truncate	ovate, apex acuminate and mucronate, base truncate	oblong-ovate, apex obtuse, base rounded
Corolla length	3.5–6 cm	4–5 cm	c. 5.5 cm	3–4 cm
Corolla indumentum (exterior)	pubescent	pubescent	glabrous	Sparsely pubescent

## Taxonomy

### Ipomoea attenuata

Taxon classificationPlantaeSolanalesConvolvulaceae

J.R.I.Wood & Scotland sp. nov.

urn:lsid:ipni.org:names:77166175-1

Figure 1

#### Diagnosis.

*Ipomoea attenuata* has been generally treated in herbaria as *I. campestris* because of the similar leaves and the pubescent exterior of the corolla, but is readily distinguished by the distinctive ovate sepals, 11–14 mm long, with truncate base and long attenuate apex. Additionally, the inflorescence is of elongate complex cymes, somewhat racemose in form and with distinctive persistent linear-lanceolate bracteoles. This contrasts with *I. campestris*, which has oblong-ovate, acute sepals 8–11 mm long, clearly narrowed to the base. The inflorescence of *I. campestris* is short, usually less than 5 cm long, and consists of shortly pedunculate cymes, these often reduced to single flowers, the bracteoles caducous (Table 1).

**Figure 1.**
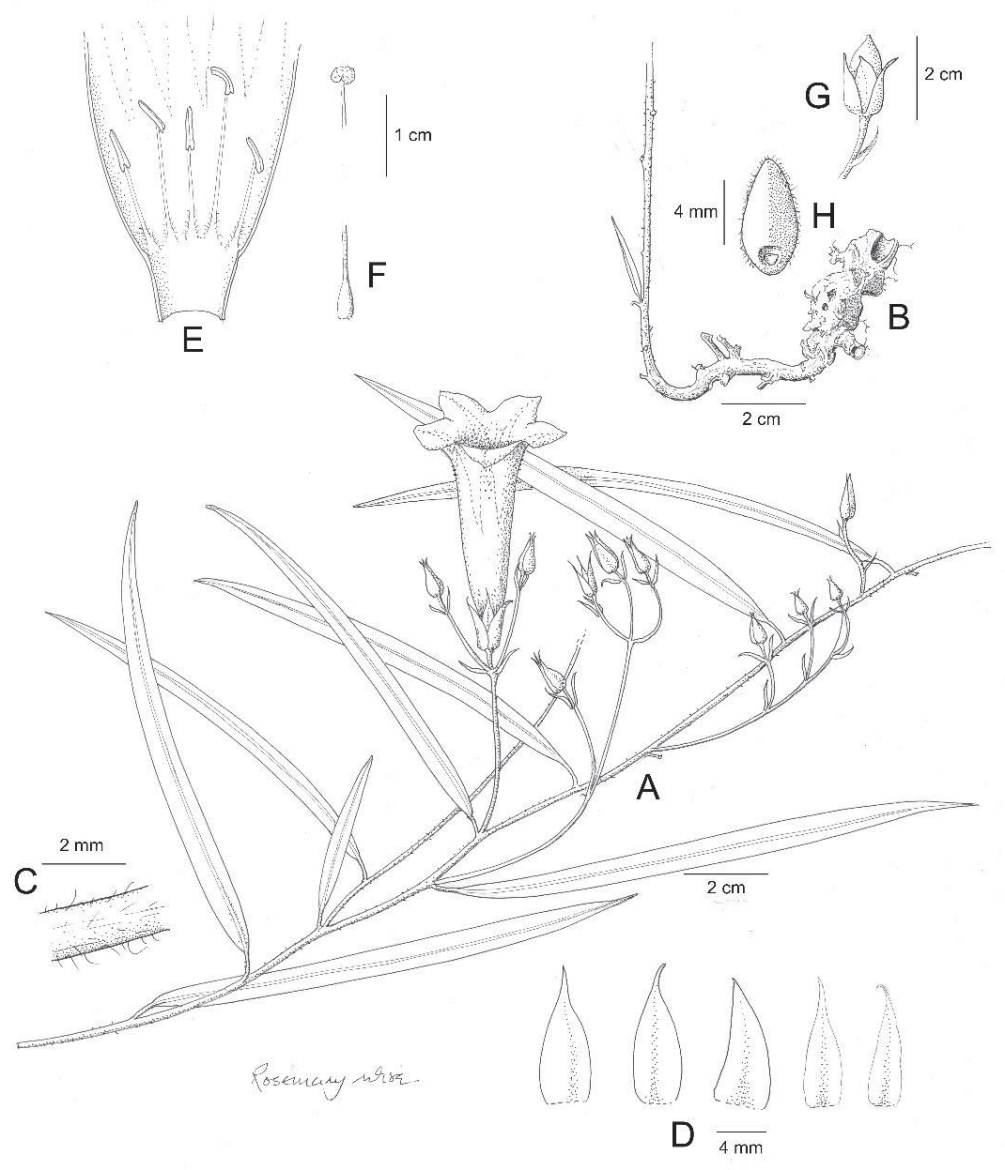
*Ipomoea attenuata* **A** habit **B** xylopodium **C** section of stem showing indumentum **D** sepals outermost (left) to innermost (right) **E** corolla opened out to show stamens **F** ovary and style **G** capsule **H** seed. Drawn by Rosemary Wise **A–E** from *H.S. Irwin et al.* 11019 **F–H** from *M. Mendoza et al.* 4802.

#### Type.

BRAZIL. Distrito Federal, Loc. Gama, BR 60, ca. 8.2 km do Tevo, DF-180 SO, disturbed campo sujo, dispersed locally, 15.5756°S 48.1059°W, 1030 m, 26 Feb. 2015, *M. Mendoza, J.B.A. Brugel, A.A. Santos, T. Reis & T.K.M. Arquelão* 4802 (holotype UB, isotypes CEN, K).

#### Description.

Perennial herb; rootstock a woody xylopodium; stems up to 80 cm long, 2 mm diam., decumbent, weakly ascending or, fide field notes, climbing, pubescent with relatively long, often twisted, spreading and appressed hairs. Leaves shortly petiolate, 4–10 × 0.3–0.7 cm, narrowly oblong, entire, apex acute and shortly mucronate, base cuneate, both surfaces thinly pubescent but more densely so abaxially; petioles 3–7 mm long, pubescent. Inflorescence of lax, compounded axillary cymes from the middle and upper leaf axils; cymes up to 15 cm long, rather narrow, diminishing in size upwards, irregularly racemose in form; peduncle 2–7 cm long, often extending into a rhachis, pubescent; primary bracteoles foliose, 9–12 × 1–3 mm, linear, acuminate, persistent; secondary peduncles 0.5–2 cm long, thinly pubescent; ultimate bracteoles 4–7 × 0.5–1 mm, linear-lanceolate, finely acuminate to attenuate, persistent; pedicels very short, 3–5 mm long, a few scattered hairs present; calyx ovate in outline; sepals subequal, 11–14 × 4–5 mm, ovate with distinct truncate base and long-attenuated acuminate apex, glabrous, the inner sepals very slightly longer than outer; corolla funnel-shaped, pink or reddish-purple, 4–5 cm long, pubescent on the midpetaline bands, limb c. 2.5–3 cm diam., shallowly lobed; stamens glabrous except at the base, unequal, longer 1.7–1.8 cm, shorter c. 1 cm, anthers linear, c. 3 mm long; ovary bilocular, glabrous; style glabrous, stigma bilobed. Capsule 13–15 × 8 mm, ovoid, glabrous; seeds 7 × 3.5 mm, ellipsoid, blackish-brown, glabrous except for pubescence along the angles.

#### Distribution and habitat.

**Figure 2.**
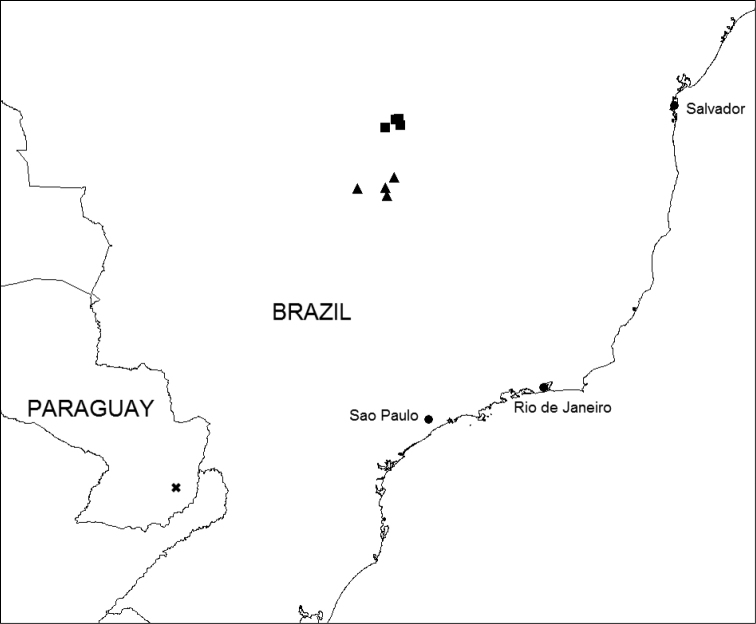
Map of Eastern Brazil and Paraguay showing distribution of *Ipomoea attenuata* (▲), *I. dasycarpa* (■) and *I. dolichopoda* (✖).

BRAZIL. Endemic to the Distrito Federal and Goiás State, where it appears to be a rare species of cerrado. Figure 2.

#### Additional collections seen.

Goiás: Samambaia, Rio Corumbá, *E.P. Heringer* 11283 (NY). Goiás: Mun. Luziania, Santo Antonio do Descoberto, *R.C. Mendonça* 93 (IBGE, NY). Goiás: Serra dos Pireneus, c. 20 km S of Corumbá de Goiás, *H.S. Irwin et al.* 11019 (NY).

#### Conservation status.

This species has been found in four separate locations. Two of the four collections are of apparent unicates and the only collection for which any details are available is the type. The field notes of this collection describe the plant as dispersed in campo sujo. *Ipomoea attenuata* is, therefore, apparently rare but relatively widely distributed. (Fig. 2). It has an area of occupancy of approximately 16.000 km², thus indicating Endangered status (EN) within IUCN guidelines (2012) but should probably be treated as Data Deficient (DD) until the populations of this species can be carefully evaluated.

#### Etymology.

The epithet “*attenuata*” refers to the attenuate tips of the sepals and bracteoles.

#### Notes.

Although we have not been able to sequence this species, *I. attenuata* probably belongs to a large clade of around 70 species almost restricted to South America, which is characterised morphologically by the pubescent exterior of the corolla and the subequal, pubescent, ovate herbaceous sepals. However, the attenuate sepal tips raise doubts about this tentative placement as this shape is atypical of species in this clade.

The form of the inflorescence (axillary cymes) combined with the oblong leaf shape strongly suggests this is essentially a decumbent species even though this is not indicated in field notes.

### Ipomoea cuscoensis

Taxon classificationPlantaeSolanalesConvolvulaceae

J.R.I.Wood & P.Muñoz sp. nov.

urn:lsid:ipni.org:names:77166176-1

Figures 3 , 4

#### Diagnosis.

*Ipomoea cuscoensis* is a distinct species because of its glabrous indumentum, 3–5-lobed leaves, large ovate-elliptic scarious-margined sepals and striking purple corolla. It is most likely to be confused with *I. peruviana* O’Donell and *I. clavata* (G. Don) Ooststr. ex J.F. Macbr., but differs from both in the purple corolla, c. 6.5 cm long, and in having all leaves 3–5-lobed. *I. peruviana* differs additionally in its much larger (10–11 cm long), very pale blue corolla and mostly entire leaves while *I. clavata* differs in its solitary blue flowers and pilose stems.

**Figure 3.**
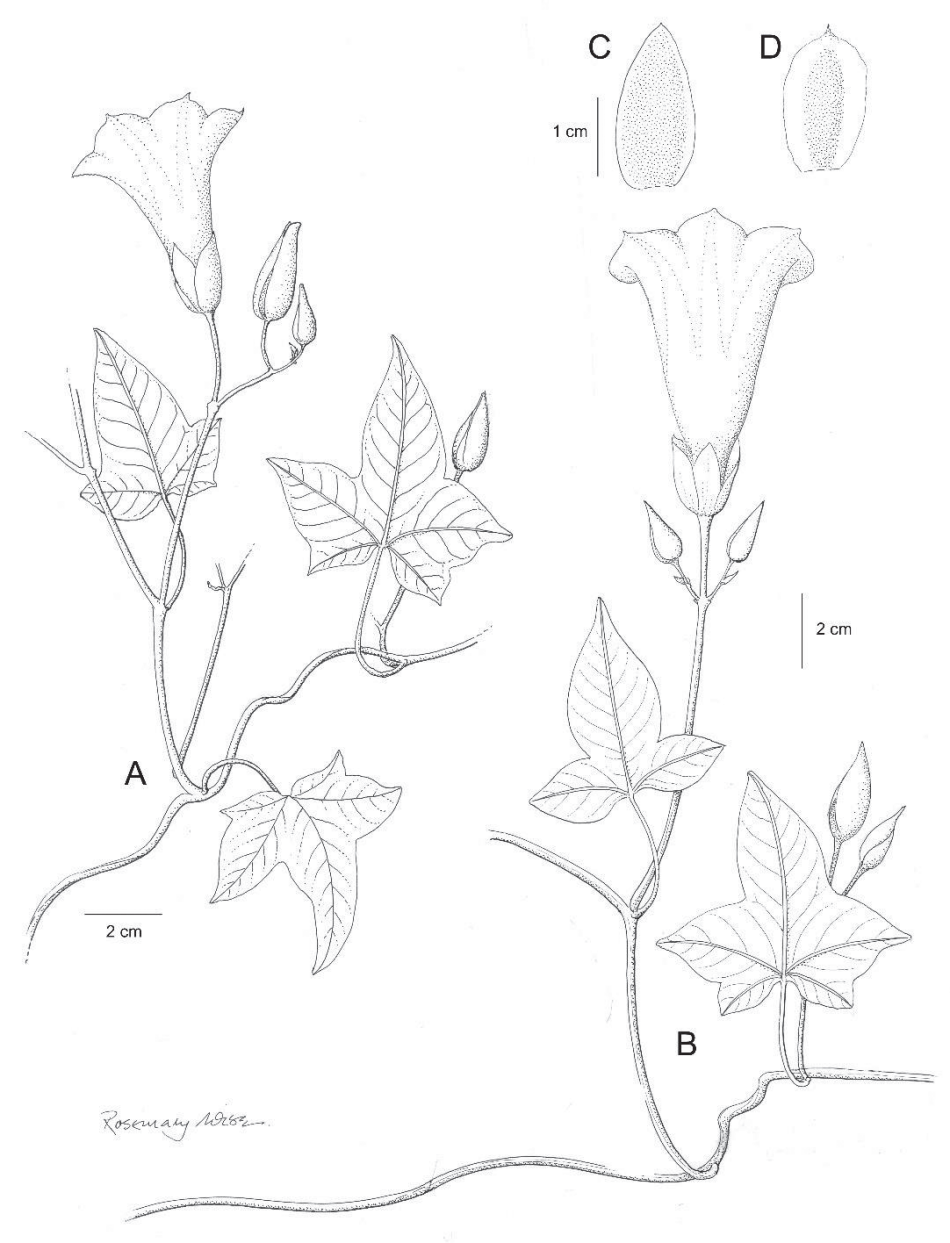
*Ipomoea cuscoensis*. **A** habit **B** habit; **C** outer sepal; **D** inner sepal. Drawn by Rosemary Wise from *Galiano et al.* 5146.

**Figure 4.**
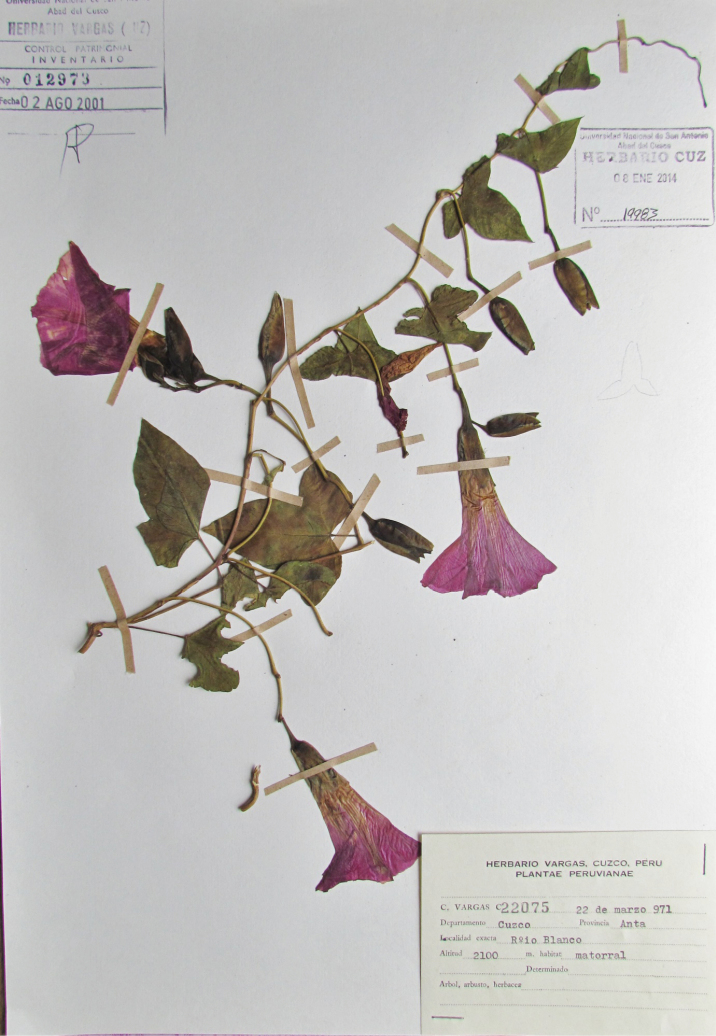
Image of *Vargas* 22075 showing the distinctive purple flowers of *Ipomoea cuscoensis*.

#### Type.

PERU. Cusco: Anta, Sisal, Limatambo, 2300 m, 15 March 1963, *C. Vargas* 14325 (holotype CUZ, isotype US).

#### Description.

Twining perennial of unknown height; stems glabrous. Leaves petiolate, 3–6 × 3–6.5 cm, 3–5-lobed, the terminal lobe larger, lobes elliptic in outline, apex acuminate to an obtuse mucronate tip, base shallowly cordate, margin weakly crenate, both surfaces glabrous, abaxially paler with prominent whitish veins; petioles 1.3–3 cm. Inflorescence of pedunculate axillary cymes with up to 7 flowers; peduncles 4–6 cm, glabrous; bracteoles caducous, not seen; pedicels 8–20 mm, glabrous; calyx narrowly ovoid, sepals somewhat unequal, outer sepals 20–22 × 10 mm, ovate to ovate-elliptic, shortly mucronate, glabrous, margins scarious; inner sepals 15 × 8 mm, ellipsoid, mucronate, the scarious margins broad; corolla 6.5 cm long, campanulate, glabrous, deep pink, limb 3–4 cm diameter. Capsule and seeds unknown.

#### Distribution and habitat.

**Figure 5.**
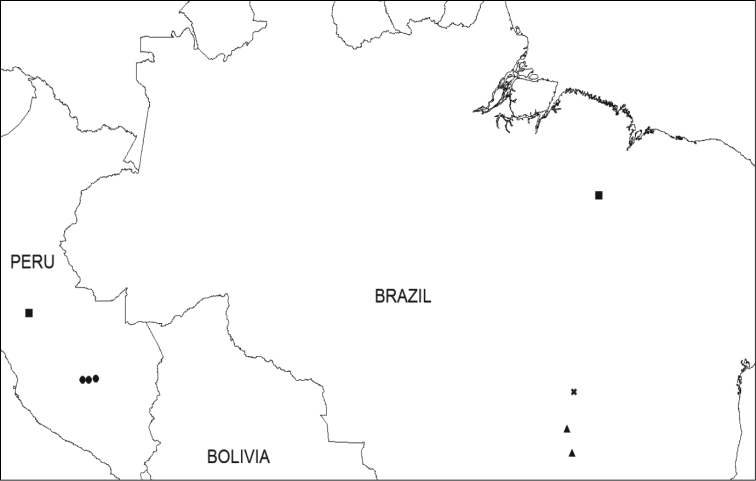
Map of equatorial South America showing the distribution of *Ipomoea cuscoensis* (●), *I. graminifolia* (✖), *I. uninervis* (▲) and *I. velutinifolia* (■).

Endemic to dry forest and scrub at 2100–2700 m in Southern PERU. Figure 5.

#### Additional collections seen.

Apurimac: Abancay, Cachora, 2700 m, 17 Jan 1950, *C. Vargas* 9083 (CUZ); Grau, bajando a Kai Ranka, 2400 m, 9 March 1946, *C. Vargas* 5826 (CUZ). Cusco: Anta, Río Blanco, 2100 m, 22 March 1971, *C. Vargas* 22075 (CUZ); Mollepata, 2710 m, 3 May 2003, *W. Galiano et al*. 5146 (MO).

#### Conservation status.

Although we have seen five separate collections of this species, they all come from a restricted area of Peru and the labels provide no information about the plant’s frequency. It should probably be treated as a “black star” species within the classification of Hawthorne and Marshall (2016) and be classified as Endangered (EN) within IUCN (2012) guidelines because of an area of occupancy of < 12, 000 km², based on an analysis using GEOCAT. However, these classifications should be treated as provisional until a full field evaluation is carried out.

#### Etymology.

The epithet “*cuscoensis*” refers to the Cusco region to which this species is endemic.

#### Notes.

Phylogenetic analysis using *ITS* sequences (unpublished data) indicates that this species belongs to a small clade, which includes *I. lindenii* M Martens & Galeotti, *I. corymbosa* (L.) Roth ex Roem. & Schult. and *I. clavata*, all of which are characterised by their relatively large sepals. It has been confused with *I. peruviana* both in the herbarium and also by Wood and Scotland (2017b), partly because *I. peruviana* is poorly known and partly because images of *I. cuscoensis* have only recently become available to us after Muñoz’s visit to CUZ in April 2017. These make clear the very distinctive flowers of the new species and confirm that it grows at the relatively high altitude of 2100 to 2710 m, at a much higher elevation than the lowland *I. peruviana*.

### Ipomoea dasycarpa

Taxon classificationPlantaeSolanalesConvolvulaceae

J.R.I.Wood & Scotland sp. nov.

urn:lsid:ipni.org:names:77166177-1

Figure 6

#### Diagnosis.

*Ipomoea dasycarpa* is close to *I. verbasciformis* (Meisn.) O’Donell but is distinguished by the larger dimensions of the leaves (2.5–11 × 1–3.5 cm, not 3–5.5 × 1–2.5 cm), bracteoles (15–20, not 5–12 mm long) and sepals (14–18 × 5–8, not 10–12 × 5 mm), by the strongly mucronate leaves, acuminate, submucronate (not obtuse) sepals and the comose (not glabrous) ovary.

**Figure 6.**
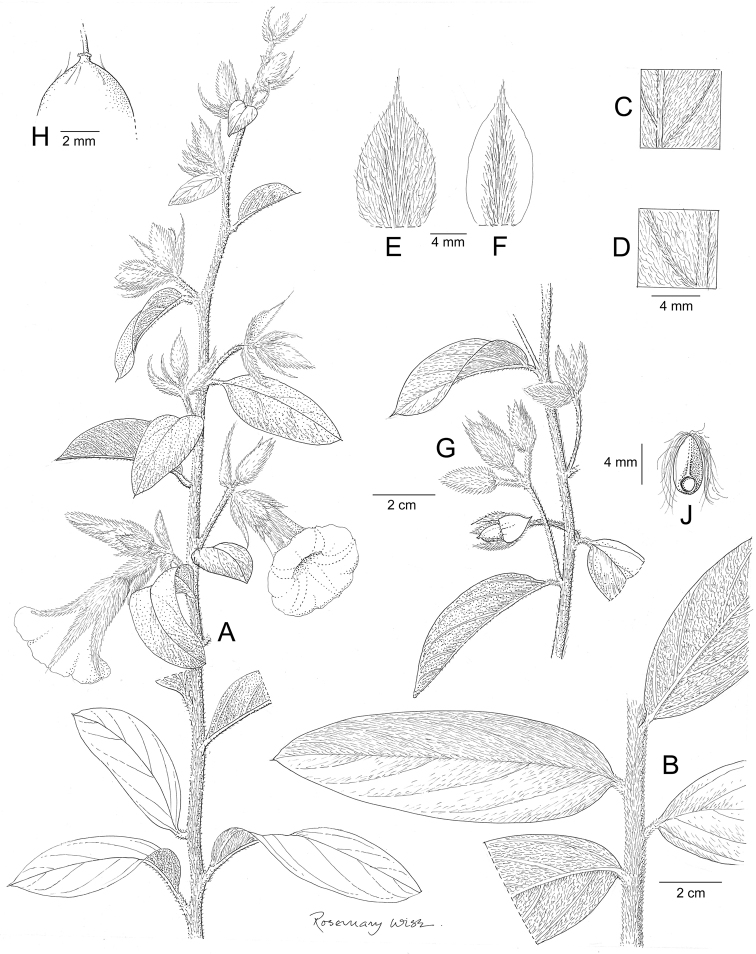
*Ipomoea dasycarpa*. **A** habit **B** leaves and stem **C** adaxial leaf surface **D** abaxial leaf surface **E** outer sepal **F** inner sepal **G** fruiting inflorescence with fallen bracteoles **H** apex of capsule **J** seed. Drawn by Rosemary Wise: **A–D** from *J.R. Pirani et al.* 1715, **E–F, H, J** from *H.S. Irwin et al.* 32875, **G** from *H.S. Irwin et al.* 24946.

#### Type.

BRAZIL. Goiás, P.N. Chapada dos Veadeiros, ca. 1100 m, perto da sede do parque, *J.R. Pirani, R.M. Harley, B.L. Stannard, A. Furlan & C. Kameyama* 1715 (holotype SPF, isotype K000944456).

#### Description.

Erect perennial subshrub to 1 m, rootstock unknown, presumably a xylopodium, stem densely tomentose with white hairs. Leaves very shortly petiolate, 2.5–11 × 1–3.5 cm, oblong to narrowly-oblong-elliptic, margin entire, base cuneate, apex acute, mucronate, the mucro 1.5–2 mm long, often bent, adaxially green, tomentose, abaxially whitish, tomentose, veins prominent; petioles 2–5 mm, tomentose. Inflorescence terminal formed of shortly pedunculate, 3-flowered cymes arising in the axils of the reduced uppermost leaves; peduncles 1–5.5 cm, grey-tomentose; lower bracteoles 15–20 × 4–7 mm, foliose, elliptic, acuminate to a fine point and ±mucronate, tomentose, persistent; upper bracteoles similar, but slightly smaller; pedicels 0–11 mm, tomentose; sepals subequal, outer 15–18 × 6–8 mm, ovate, acuminate, submucronate, tomentose, inner 14–15 × 5–7 mm, tomentose with broad glabrous margins; corolla 4.5–5 cm, funnel-shaped, pink, tomentose in bud, limb c. 4 cm diameter, entire. Capsule 9 × 5 mm, ovoid, muticous, comose with shaggy, somewhat deciduous hairs; seeds 6 × 3 mm, glabrous apart from the fine white marginal hairs c. 5–6 mm long.

#### Distribution and habitat.

BRAZIL. Endemic to relatively high altitudes between 1000 and 1250 m in the Chapada dos Veadeiros in Goiás, apparently growing in rocky cerrado. Figure 2.

#### Additional collections seen.

Goiás: Chapada dos Veadeiros, c. 20 km W of Veadeiros, *H.S. Irwin et al*. 12407 (FTG114226); 10 km S of Alto do Paraíso, *H.S. Irwin et al*. 24946a (FTG114228); 18 km N of Alto do Paraíso, *H.S. Irwin et al*. 32875 (FTG114227); Fazenda de Sao Bento, *Glaziou* 21786 (P).

#### Conservation status.

This species has only been found on five occasions, all in the Chapada dos Veadeiros region, indicating this species is very localised in its distribution. Apparently there are duplicates of only one of these collections, suggesting the species is not common in any of the places where it has been found. Field notes are minimally informative. It should probably be treated as Data Deficient (DD) within IUCN (2012) guidelines until the populations can be carefully evaluated. All recorded locations apparently lie within the Chapada dos Veadeiros National Park, which enjoys legal protection.

#### Etymology.

The epithet “*dasycarpa*” refers to the comose ovary and capsule.

#### Notes.

Molecular sequencing using *ITS* (unpublished data) shows that *I. dasycarpa* belongs to a large clade of around 70 species almost restricted to South America, which is characterised morphologically by the pubescent exterior of the corolla and the subequal, pubescent, ovate herbaceous sepals. The comose (not glabrous) ovary is an especially interesting character as hirsute capsules are rare in *Ipomoea* and found outside the Batatas clade in only a few species such as *I. sidifolia* Schrad. and *I. velutinifolia* described below. None of these appear to be related to or resemble *I. dasycarpa*.

### Ipomoea dolichopoda

Taxon classificationPlantaeSolanalesConvolvulaceae

J.R.I.Wood & R.Degen sp. nov.

urn:lsid:ipni.org:names:77166178-1

Figure 7

#### Diagnosis.

*Ipomoea dolichopoda* resembles *I. attenuata* in the oblong, shortly petiolate leaves, persistent bracteoles and ovate sepals with a distinct truncate base and attenuate apex but differs in the long, stiff white hairs, 1–2 mm in length, which are scattered over all vegetative parts, the very short primary peduncles combined with very long secondary peduncles, the pilose outer sepals and the glabrous exterior of the corolla. Table 1.

**Figure 7.**
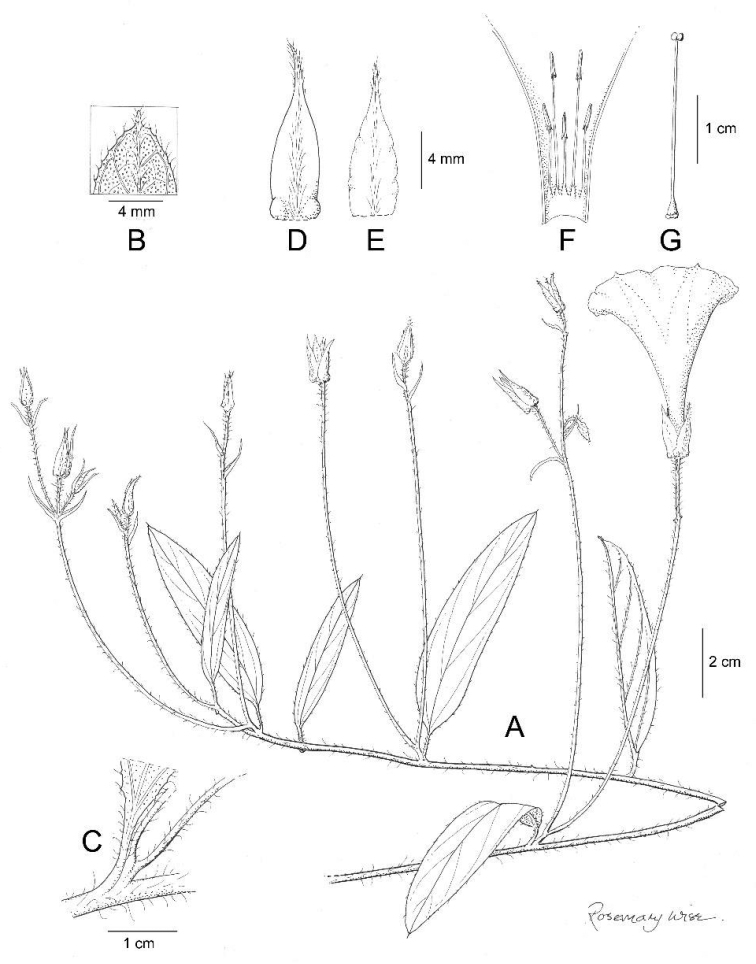
*Ipomoea dolichopoda*. **A** habit **B** leaf apex **C** leaf base, showing peduncle and inflorescence **D** Outer sepal **E** inner sepal **F** corolla opened out to show stamens **G** ovary and style. Drawn by Rosemary Wise from *I. Basualdo* 002775.

#### Type.

PARAGUAY. Caazapá, Castor Cue, 26°10'S, 55°20'W, *I. Basualdo* 002775 (holotype FCQ, isotype MO).

#### Description.

Trailing herb, probably perennial; stems thinly pilose with white hairs. Leaves petiolate, 4–6.5 × 0.8–1.5 cm, slightly oblique, oblong, base cuneate, apex obtuse and mucronate, margins ciliate, adaxially glabrous, punctate, abaxially pilose on the veins; petioles 7–8 mm, thinly pilose. Inflorescence of pedunculate axillary cymes with 1–4 flowers borne on long secondary peduncles; primary peduncles 0.3–1.2 cm; secondary peduncles 7–12 cm, thinly pilose; bracteoles 9–12 × 1 mm, filiform, persistent till anthesis; pedicels 8–15 mm, pilose; sepals 10–14 × 3–4 mm, ovate, finely acuminate to a mucronate apex, base rounded to truncate, outer sepals pilose except at margins, inner sepals slightly shorter with glabrous, scarious margins; corolla c. 5.5 cm long, broadly funnel-shaped, glabrous even in bud, pink, limb c. 3.5 cm diam.; stamens unequal, filaments, glabrous except at base, longer pair c. 1.8 cm, shorter c. 1 cm, anthers linear, 4 mm, included; ovary presumably bilocular; stigma bi-globose. Capsule and seeds unknown.

#### Distribution and habitat.

PARAGUAY. Only known from the type collection which was found in “praderas,” presumably some kind of cerrado grassland in eastern Paraguay. Figure 2.

#### Conservation status.

Field notes give no data about the frequency of this species and in the absence of other collections or any information about threats to its habitat, it can only be classified as Data Deficient (DD) within IUCN guidelines. It would be treated as a “black star” species within the classification of Hawthorne and Marshall (2016), but again this must be considered a provisional classification as no systematic search has been made for the species at the type locality or in other suitable habitats, although it must be presumed to be rare.

#### Etymology.

The epithet *dolichopoda* meaning “long-stalked” refers to the exceptionally long secondary peduncles of the inflorescence.

#### Notes.

This species has a strong superficial resemblance to *I. attenuata*, described above. Both species have somewhat similar oblong, shortly petiolate leaves and ovate sepals with a distinct truncate base and acuminate apex. The persistent bracteoles are also somewhat similar. *Ipomoea dolichopoda*, however, can be distinguished at first glance by the long white hairs which are scattered over all vegetative parts including the pedicels and sepals. It is also distinct in the very short primary peduncles combined with the very long secondary peduncles, a combination that in our experience is unique in *Ipomoea*. The glabrous exterior of the corolla is another distinguishing feature which raises doubts about the relationship of the two species and serves to distinguish it from *I. delphinioides* Choisy, with which it has been identified.

### Ipomoea ensiformis

Taxon classificationPlantaeSolanalesConvolvulaceae

J.R.I.Wood & Scotland sp.nov.

urn:lsid:ipni.org:names:77166179-1

Figure 8

#### Diagnosis.

*Ipomoea ensiformis* somewhat resembling *I. campestris* but differs in a range of characters. It is a prostrate, glabrescent (pubescent only on young parts) plant, the leaves distinctly petiolate and terminating in an obtuse apex. The sepals are glabrous, < 8 mm long and the corolla is smaller at 3–4 cm long and relatively widely funnel-shaped. In contrast, *I. campestris* is usually erect, pubescent to pilose in all vegetative parts and the leaves are subsessile, acute and mucronate. The sepals are 8–11 mm long, the exterior pubescent while the corolla is about 5.5 to 6 cm in length. Table 1.

**Figure 8.**
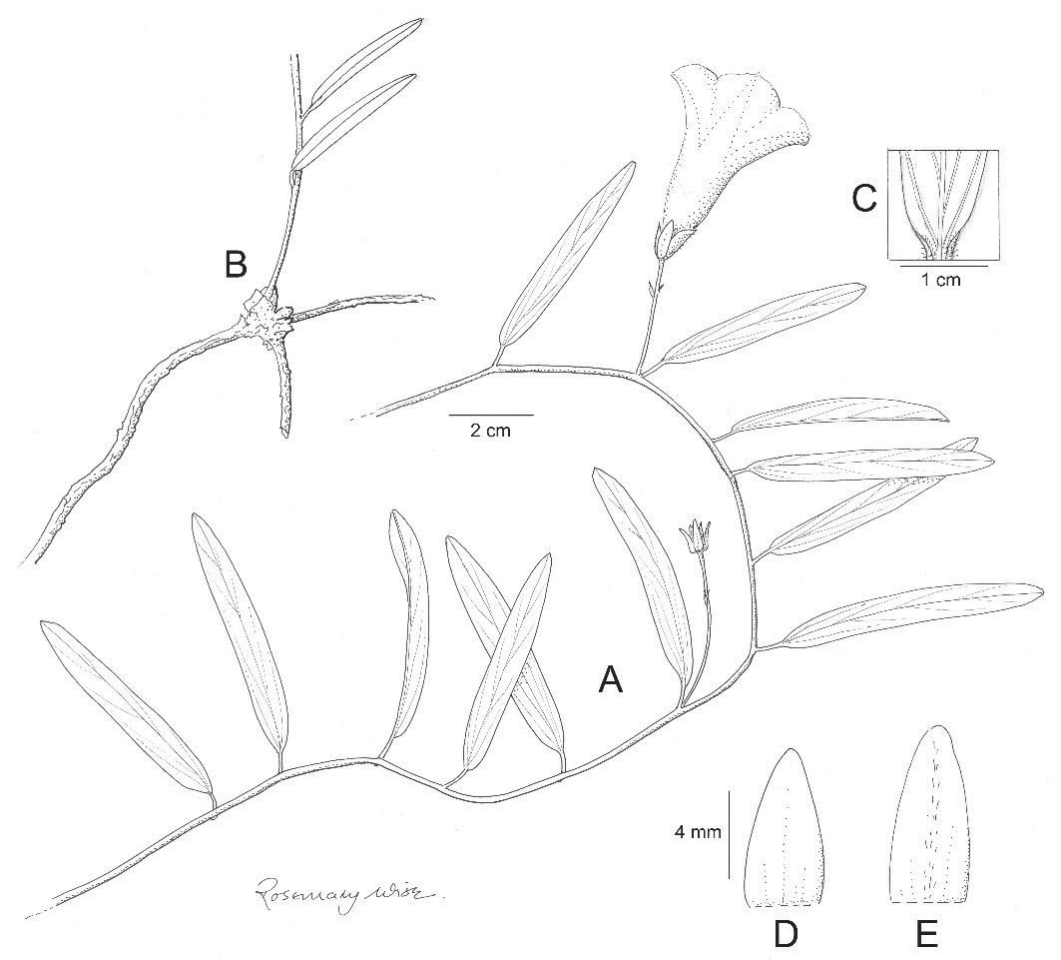
*Ipomoea ensiformis*. **A** habit **B** rootstock **C** base of young leaves showing indumentum **D** Outer sepal **E** inner sepal. Drawn by Rosemary Wise from *Gates & Estabrook* 4.

#### Type.

Brazil, Goias, 5 km Alto Paraiso, Chapada dos Veadeiros, 1450 m, 24 Jan 1979, *Gates & Estabrook* 4 (holotype UB62303, isotypes MICH, RB00263006).

#### Description.

Procumbent perennial herb, stems thinly pubescent, to 50 cm; rootstock a knotted woody xylopodium. Leaves shortly petiolate, 2–6 × 0.3–1.2 cm, oblong to oblong-lanceolate, base rounded, apex subacute to obtuse, very shortly mucronate, margin entire to undulate, glabrescent, the very young leaves pubescent; petioles 1–4 mm, puberulent. Inflorescence of solitary (rarely paired), axillary flowers borne on slender peduncles; peduncles 1.4–3.2 cm, slender, puberulent; bracteoles 3 × 1 mm, ovate, acuminate, relatively persistent; pedicels 5–6 mm, thinly puberulent; sepals subequal, outer 6–7 × 2.5–3 mm, oblong-ovate, obtuse, glabrous, inner similar but narrowly oblong-ovate, 7–8 mm long, abaxial surface sparsely pubescent centrally; corolla 3–4 cm, pink, very sparsely pubescent on midpetaline bands, funnel-shaped, limb 3.5 cm diameter. Capsule and seeds not seen.

#### Distribution and habitat.

**Figure 9.**
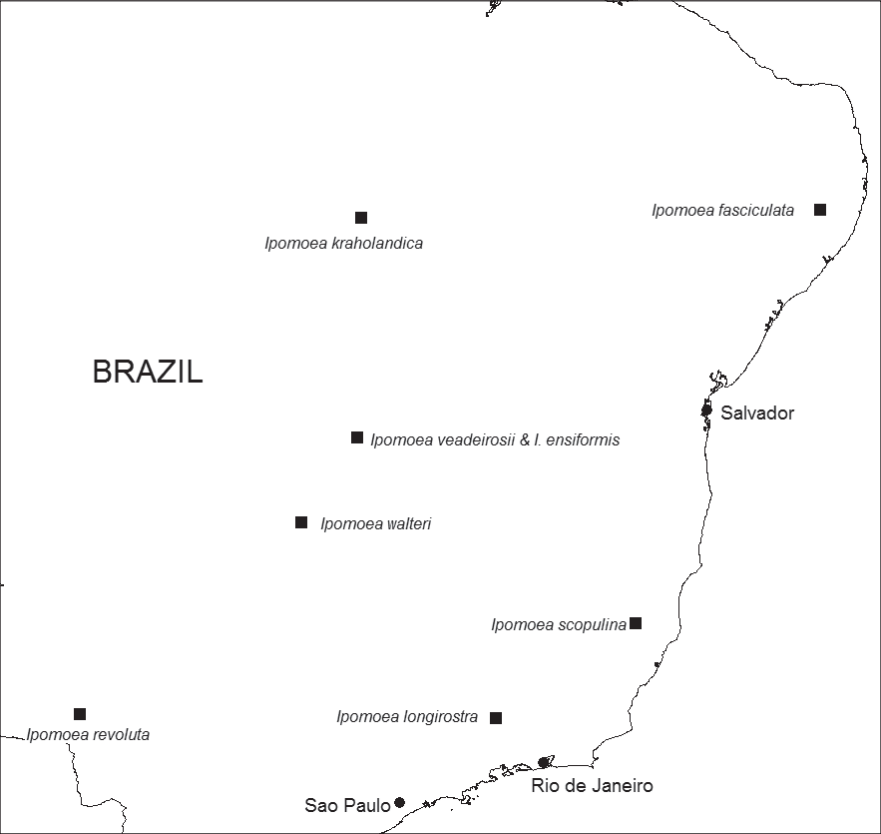
Map of Eastern Brazil showing distribution of *I. ensiformis*, *I. fasciculata*, *I. kraholandica*, *I. longirostra*, *I. revoluta*, *I. scopulina*, *I. veadeirosii*, and *I. walteri*.

BRAZIL. Endemic to Goiás State where it is only known from the type collection. It is one of several *Ipomoea* species which are apparently restricted to the Chapada dos Veadeiros and, like *I. graminifolia*, was found at 1450 m, an exceptionally high altitude in the Brazilian planalto. Figure 9.

#### Conservation status.

In the absence of other collections or any information about its frequency, the status of this species can only be classified as Data Deficient (DD) within IUCN (2012) guidelines. It would be treated as a “black star” species within the classification of Hawthorne and Marshall (2016), but again this must be considered as a provisional classification as no systematic search has been made for the species at the type locality or in other similar habitats in the area.

#### Etymology.

The epithet *ensiformis* refers to the shape of the leaves, which resemble small swords.

#### Notes.

This has the appearance of a nearly glabrous prostrate form of *Ipomoea campestris*. *I. campestris* is quite variable in leaf shape but is always readily distinguished by the longer, narrower corolla, which reaches 6 cm, and the conspicuous pubescent indumentum.

### Ipomoea fasciculata

Taxon classificationPlantaeSolanalesConvolvulaceae

J.R.I.Wood & Scotland sp. nov.

urn:lsid:ipni.org:names:77166180-1

Figure 10

#### Diagnosis.

*Ipomoea fasciculata* is a very distinct species with no obvious affinities, distinguished by the clustered flowers forming a subcapitate inflorescence, the small, prominently mucronate, scarious glabrous sepals and the small, rostrate capsule.

**Figure 10.**
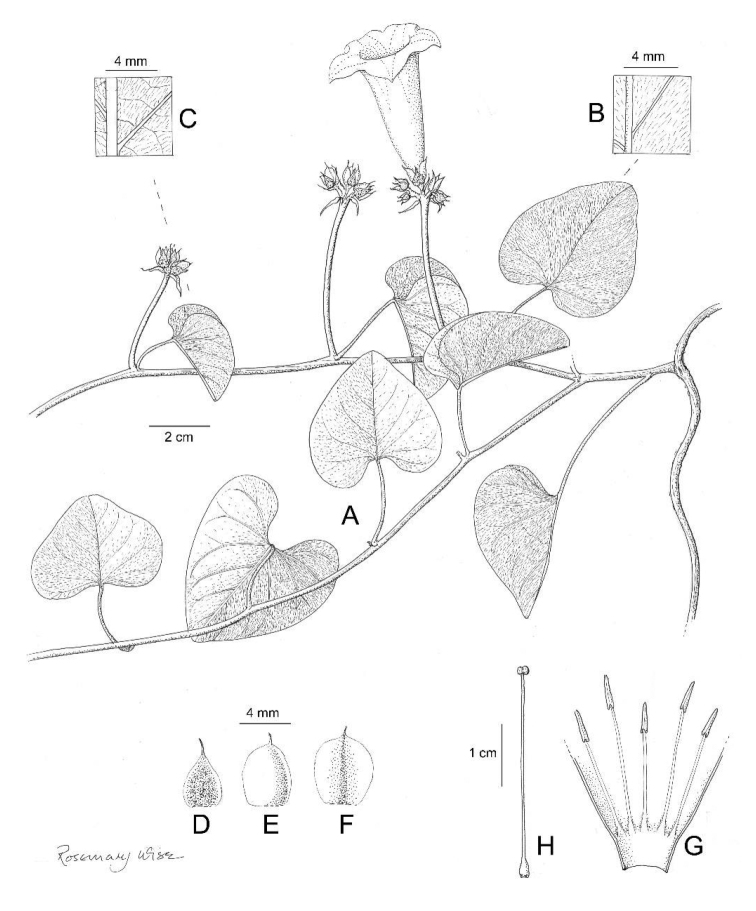
*Ipomoea fasciculata*. **A** habit **B** adaxial leaf surface **C** abaxial leaf surface **D** outer sepal **E** middle sepal **F** inner sepal **G** corolla opened out to show stamens **H** ovary, style and stigma. Drawn by Rosemary Wise from *P. Gomes et al.* 658.

#### Type.

BRAZIL, Pernambuco, Agrestina, Inselberg Pedra Cabeça de Velho, 8°23'29.7"S 36°00'37.6"W, 832 m, 22 Oct. 2007, *P. Gomes, M. Alves & B. Maciel* 658 (holotype RB00601358, isotype UFP, n.v.).

#### Description.

Climbing perennial, stem minutely puberulent, glabrescent. Leaves petiolate, 2.5–5.5 × 2.4–5 cm, ovate, cordate with rounded auricles, apex acute, margin obscurely undulate, both surfaces shortly puberulent, abaxially paler; petioles 1–4.5 cm, thinly puberulent. Inflorescence of pedunculate axillary cymes reduced to pedunculate clusters or fascicles; peduncles 3.5–5.5 cm, puberulent; bracteoles 5 × 1.5 mm, lanceolate, acuminate, scarious; pedicels 1–3 mm, pubescent; sepals slightly unequal, glabrous, outer 5–6 × 3 mm, ovate, mucronate, abaxially slightly muricate, margin scarious, inner 6–8 × 4–6 mm, oblong-elliptic, rounded and mucronate, entirely scarious except central area; corolla c. 5 cm long, pink, funnel-shaped, glabrous, limb 3 cm diameter; stamens included; longer filaments 12–15 mm, shorter c. 10 mm; ovary bilocular, style c. 2.5 cm, glabrous, stigma bi-globose. Capsule (immature) ovoid, rostrate, 5 × 3 mm, glabrous; seeds not seen.

#### Distribution and habitat.

BRAZIL. Endemic to Pernambuco State in NE Brazil and only known from the type collection. It was found growing on the Inselberg Pedra Cabeça de Velho at 832 m, presumably on granite rock. The vegetation is described in more detail by Gomes and Alves (2010), who report that, as in Bolivia (Wood 2009), plant families such as Bromeliaceae, Leguminosae and Asteraceae are common, genera such as *Croton* L. and *Chamaecrista* Moench being especially characteristic. Figure 9.

#### Conservation status.

In the absence of other collections or any information about threats to its habitat, the status of this species can only be classified as Data Deficient (DD) within IUCN (2012) guidelines. It would be treated as a “black star” species within the classification of Hawthorne and Marshall (2016), but again this must be considered a provisional classification as no systematic search has been made for the species at the type locality or in other similar habitats. Given the highly dispersed distribution of other inselberg species, it might be expected to occur in other isolated locations.

#### Etymology.

The epithet *fasciculata* refers to the clustered or bunched inflorescence, which is a salient characteristic of this species.

#### Note.

We have not been able to sequence this species and its relationships are not clear. It is very distinct because of the subcapitate inflorescence, the small, prominently mucronate, scarious glabrous sepals and the small, rostrate capsule.

Within the context of this paper this species is unique not only for the form of its inflorescence but also for having been found on a dome mountain within the Caatinga biome. Similar granite or crystalline domes are fairly common in parts of eastern Brazil but are less common in the Cerrado biome, although they are found in Mato Grosso and parts of Eastern Bolivia. Several species of *Ipomoea* have been described from these domes, notably *I. graniticola* J.R.I. Wood & Scotland and *I. chiquitensis* J.R.I. Wood & Scotland from Bolivia (Wood et al 2015), both of which were thought originally to have been pin-point endemics, but the former has been subsequently found in similar habitats in both Paraguay and Brazil, while the latter has been found in NE Brazil. It is to be hoped that *I. fasciculata* will be found on other inselbergs, perhaps at a considerable distance from the type locality.

### Ipomoea graminifolia

Taxon classificationPlantaeSolanalesConvolvulaceae

J.R.I.Wood & Scotland sp. nov.

urn:lsid:ipni.org:names:77166181-1

Figure 11

#### Diagnosis.

*Ipomoea graminifolia* resembles and is probably related to *I. procumbens* Mart. ex Choisy and *I. aequiloba* J.R.I. Wood & Scotland, being completely glabrous and having solitary flowers and subequal, lanceolate to ovate sepals. From *I. procumbens* it is distinguished by the much smaller calyx (the inner sepals oblong-lanceolate, only 7–9 × 2 mm , not 12–15 × 4–6 mm) and corolla (< 3.5 cm long, not >5.5cm long), the wiry stems, and the sessile, filiform leaves. In these dimensions and habit it is closer to *I. aequiloba* but is immediately distinguished by the simple, nor subequally 3-lobed leaves.

**Figure 11.**
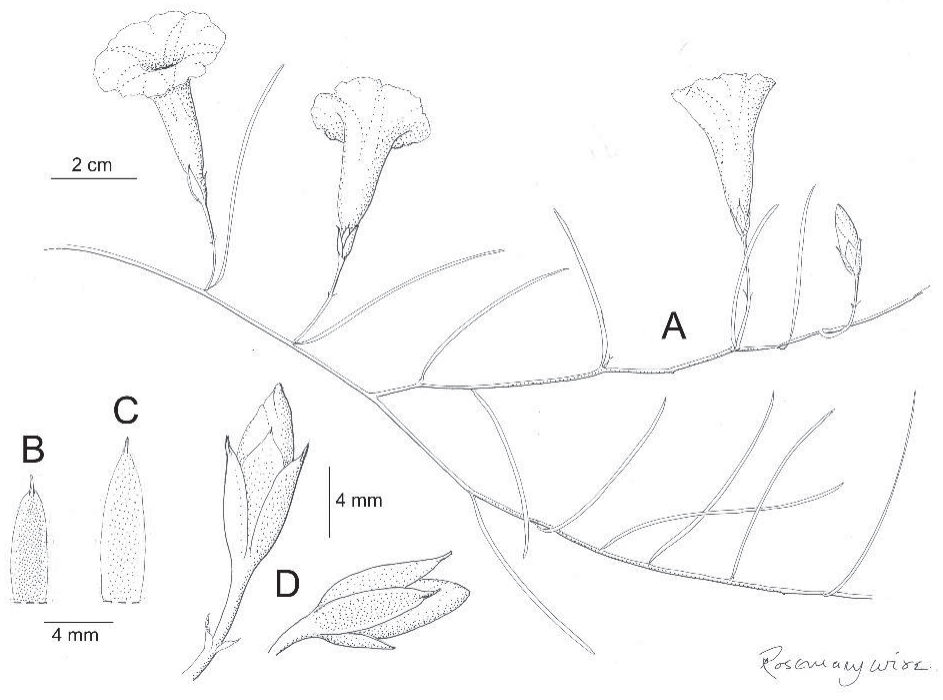
*Ipomoea graminifolia*. **A** habit **B** outer sepal **C** inner sepal **D** buds showing calyx. Drawn by Rosemary Wise from *C. Munhoz et al.* 2567.

#### Type.

BRAZIL. Goiás, Fazenda Agua Fria, Alto Paraiso de Goiás, cerca 10 km en direção a Teresina de Goiás, 14°04'217"S, 47°30'336"W, 1448 m, 20 Feb. 2001, *C. Munhoz, N. Rodrigues & K.M.O. Ramos* 2567 (holotype MO-5948847, isotypes not found).

#### Description.

Completely glabrous, slender, probably clambering perennial herb, stems thin, wiry, slightly woody. Leaves sessile, 2.5–5.5 × 0.05–0.1 cm. linear-filiform, acute, minutely apiculate. Inflorescence of solitary axillary flowers; peduncles 8–18 mm; bracteoles deltoid, 1 mm long, caducous; pedicels 6–8 mm, thickened upwards; sepals unequal, outer 5–6 × 2 mm, broadly lanceolate, acute and mucronate, margin narrow, scarious; inner 7–9 × 2 mm, oblong-lanceolate, acute, margins broad, scarious; corolla 3–3.5 cm long, funnel-shaped, pink, glabrous, limb 2.5–3 cm diameter, undulate, the midpetaline bands ending in acute points; stamens included; style very short, c. 1.2 cm, stigma bi-globose. Capsule and seeds unknown.

#### Habitat and Distribution.

BRAZIL. High altitude endemic of campo limpo úmido, only known from the type collection in Goiás, where it was collected in or near the Chapada dos Veadeiros National Park. The recorded altitude of 1400 m is unusually high for an *Ipomoea* species in Brazil. Figure 5.

#### Etymology.

The epithet *graminifolia* refers to the characteristic grass-like leaves of this species.

#### Conservation status.

In the absence of other collections or any information about threats to its habitat, the status of this species can only be classified as Data Deficient (DD) within IUCN guidelines. It would be treated as a “black star” species within the classification of Hawthorne and Marshall (2016), but again this must be considered as a provisional classification as no systematic search has been made for the species at the type locality or in other suitable habitats, although it must be presumed to be rare.

### Ipomoea kraholandica

Taxon classificationPlantaeSolanalesConvolvulaceae

J.R.I.Wood & Scotland sp. nov.

urn:lsid:ipni.org:names:77166182-1

Figure 12

#### Diagnosis.

*Ipomoea kraholandica* is a very distinct species unlike any other known to us because of the solitary flowers with suppressed peduncles, the narrowly lanceolate, pubescent sepals and the apparently unique lamina, which is essentially 3-lobed, the middle lobe lanceolate to oblong-lanceolate, the two laterals bifurcate or trifurcate with the lower lobe curved backwards whereas the upper and middle lobes (when present) are bent forward.

#### Type.

BRAZIL. Tocantins, Mun. Itacajá, Reserva Indígena Krahó, Aldea Pedra Branca, 9 May 2000, *A.A. Santos, A. Reatto, E. de Souza Martins, L. Rovênia, M. de Andrade & L. Moreira Rodrigues* 719 (holotype CEN).

#### Description.

Slender twining herb of unknown height; stems glabrous. Leaves petiolate, 2–3.5 × 1–3 cm, 3-lobed with the central lobe lanceolate, entire, the lateral lobes 2–3-lobed, the first and second lobes bent forwards and the third lobe bent backwards, base truncate, apex finely acuminate, both surfaces glabrous; petioles 0.7–2 cm. Inflorescence of solitary, axillary flowers; peduncles very short, 0–3 mm, thinly pubescent; bracteoles 1–3 mm, relatively persistent, thinly ciliate; pedicels 6–12 mm, thickened upwards, pubescent; sepals subequal, 11–12 × 1.5–2.5 mm, narrowly lanceolate, finely acuminate, mucronate, outer pubescent, inner pubescent with broad glabrous margins; corolla c. 2.5 cm long, funnel-shaped, pink, glabrous, midpetaline bands terminating in a prominent tooth, limb c. 2.5 cm diameter; filaments glabrous except for glandular-pilose base, longer pair 7–8 mm, shorter 4–5 mm; ovary bilocular, glabrous; style 8 mm, glabrous; stigma bi-globose. Capsule 10 × 5 mm, ovoid, glabrous; seeds 4, 5 × 2 mm, dark grey, minutely tomentellous.

#### Distribution.

BRAZIL. Only known from the type locality in Tocantins. Locally abundant in disturbed ground on sand. Figure 9.

#### Conservation status.

Field notes record this plant as “locally abundant” but in the absence of other collections or any information about threats to its habitat, it can only be classified as Data Deficient (DD) within IUCN (2012) guidelines. It would be treated as a “black star” species within the classification of Hawthorne and Marshall (2016), but again this must be considered a provisional classification as no systematic search has been made for the species at the type locality or in other suitable habitats, although it must be presumed to be rare.

#### Etymology.

The epithet *kraholandica* refers to Reserva Indígena Krahó, where this species was found.

#### Note.

**Figure 12.**
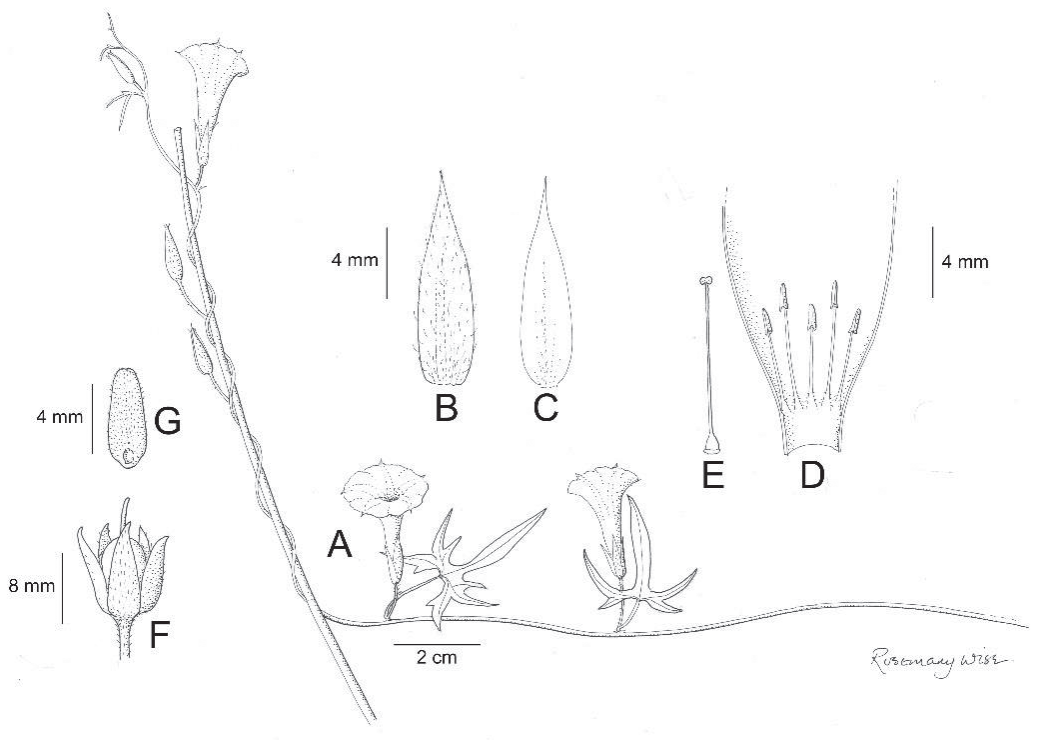
*Ipomoea kraholandica*. **A** habit **B** outer sepal **C** inner sepal **D** corolla opened out to show stamens **E** ovary and style **F** capsule and calyx **G** seed. Drawn by Rosemary Wise from *A.A. Santos et al* 719.

Molecular studies using *ITS* (unpublished data) place this species in a well-supported clade of mainly Brazilian species with *I. bahiensis* Willd. ex Roem. & Schult., *I. squamosa* Choisy, *I. eriocalyx* (Choisy) Meisn. and *I. acanthocarpa* (Choisy) Aschers. & Schweinf., and more distantly with *I. imperati* (Vahl) Griseb. and *I. longeramosa* Choisy. It does not, however, resemble any of these species except perhaps *I. longeramosa*, which differs in its cream-coloured flowers, longer peduncles 2–5 cm in length and quite different leaf shape. The leaf shape of *I. kraholandica* is especially unusual. The lamina is essentially 3-lobed, the middle lobe lanceolate to oblong-lanceolate, the two laterals bifurcate or trifurcate, the lower lobe curved backwards whereas the upper and middle (when present) lobes are bent forward (Figure 12). The seeds are shortly hirsute, best described as tomentellous.

### Ipomoea longirostra

Taxon classificationPlantaeSolanalesConvolvulaceae

J.R.I.Wood & Scotland sp. nov.

urn:lsid:ipni.org:names:77166183-1

Figure 13

#### Diagnosis.

*Ipomoea longirostra* is probably related to and certainly resembles *I. procumbens* Mart. ex Choisy in being completely glabrous and with solitary flowers but is distinguished by its ovate-deltoid, basally truncate leaves borne on slender pedicels, and the ovate-elliptic, obtuse to rounded, not lanceolate to ovate, acute to acuminate sepals. The strongly rostrate capsule is also distinct.

#### Type.

BRAZIL. Minas Gerais, Lima Duarte, P.N. Estadual do Ibitipoca, prov. Rio do Salto, 21°42'80"S, [43°47'W] (longitude missing from label), 1200 m, 9 March 2003, fl., fr., *R.C. Forzza, L.C.S. Assis. J.G. Jardim, R. Lima, L. Menini Neto, E. Lucas, B.R. Silva, S. Edwards & D. Zappi* 3031 (holotype RB00552260; isotypes K, NY)

**Figure 13.**
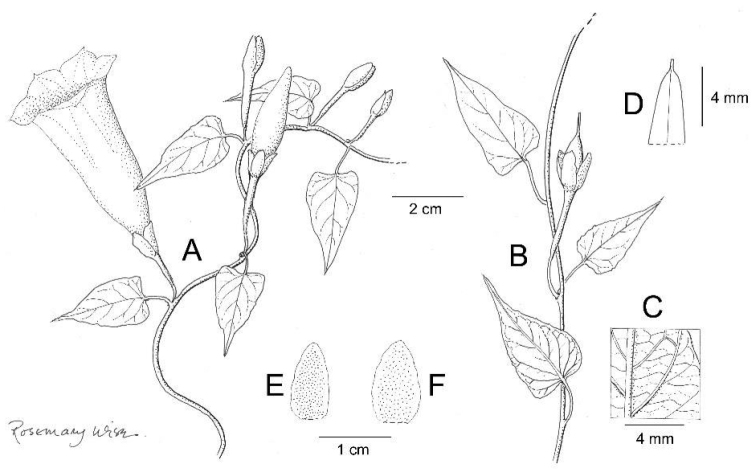
*Ipomoea longirostra*. **A** habit showing inflorescence **B** habit showing rostrate capsule **C** abaxial leaf surface **D** leaf apex **E** outer sepal **F** inner sepal. Drawn by Rosemary Wise from *R.C. Forzza et al.* 303.

#### Description.

Twining perennial of unknown height, glabrous in all vegetative parts. Leaves petiolate, 3–4 × 1.3–2.2 cm, deltoid, finely acuminate, shortly mucronate, base truncate to cordate with rounded auricles, margin denticulate, abaxially paler with prominent veins; petioles very slender, curved, 9–17 mm. Inflorescence of solitary, pedunculate, axillary flowers; peduncles 10–15 mm; bracteoles caducous, not seen; pedicels noticeably stouter than peduncles 12–15 mm; sepals subequal, elliptic, glabrous, margins scarious, outer 8–11 × 4–6 mm, obtuse, inner 9–12 × 6–7 mm, rounded, usually c. 0.5 mm longer and 1 mm wider than outer sepals; corolla 5.5 cm long, pink, glabrous, funnel-shaped, limb 3–3.5 cm diameter. Capsule 13 × 6–7 mm, conical, glabrous, strongly rostrate, the apex 4–5 mm long, persistent; seeds unknown.

#### Distribution.

BRAZIL. Endemic to the area of the type locality in the Parque Nacional Estadual do Ibitipoca in Minas Gerais. Figure 9

#### Additional collections seen.

Minas Gerais, Lima Duarte, P.N. Estadual do Ibitipoca, casa da polícia florestal, 21°42'33"S, 43°53'46"W, *R.C. Forzza et al.* 2638 (RB0055171); ibid., Cachoeira dos Macacos 6 Feb. 2004, fl., *R.C. Forzza et al.* 2692 (RB00551680); ibid., prov. Rio do Salto, campo ao lado do alojamento, Nov. 2006, fl., *R.C. Forzza et al.* 4362 (NY01018831, RB00552445).

#### Conservation status.

The four collections were made at different dates from the near vicinity of each other. Field notes do not indicate the plant’s frequency and in the absence of other collections or any information about threats to its habitat, it can only be classified as Data Deficient (DD) within IUCN (2012) guidelines. It would be treated as a “black star” species within the classification of Hawthorne and Marshall (2016), but again this must be considered a provisional classification as no systematic search has been made for the species at the type locality or in other suitable habitats, although it must be presumed to be rare.

#### Etymology.

The epithet *longirostra* refers to the prominently beaked capsule.

#### Notes.

We have not been able to sequence material of this species but it is almost certainly related to *I. procumbens* under which name it was provisionally identified at Kew. Although *I. procumbens* is a quite variable species, it never has the finely petiolate, truncate leaves of *I. longirostra*. The strongly rostrate capsule of the new species is also very striking.

### Ipomoea revoluta

Taxon classificationPlantaeSolanalesConvolvulaceae

J.R.I.Wood & Scotland sp. nov.

urn:lsid:ipni.org:names:77166184-1

Figure 14

#### Diagnosis.

*Ipomoea revoluta* is almost certainly related to *I. malvaeoides* Meisn. and its allies but is distinguished from all of these by its twining (not erect) habit and distinctly petiolate leaves. Related species in which the leaves are furnished with linear leaflets, such as *I. fiebrigii* O’Donell, *I. itapuaensis* J.R.I. Wood & R. Degen and *I. theodori* O’Donell, are erect herbs with sessile or near sessile leaves.

**Figure 14.**
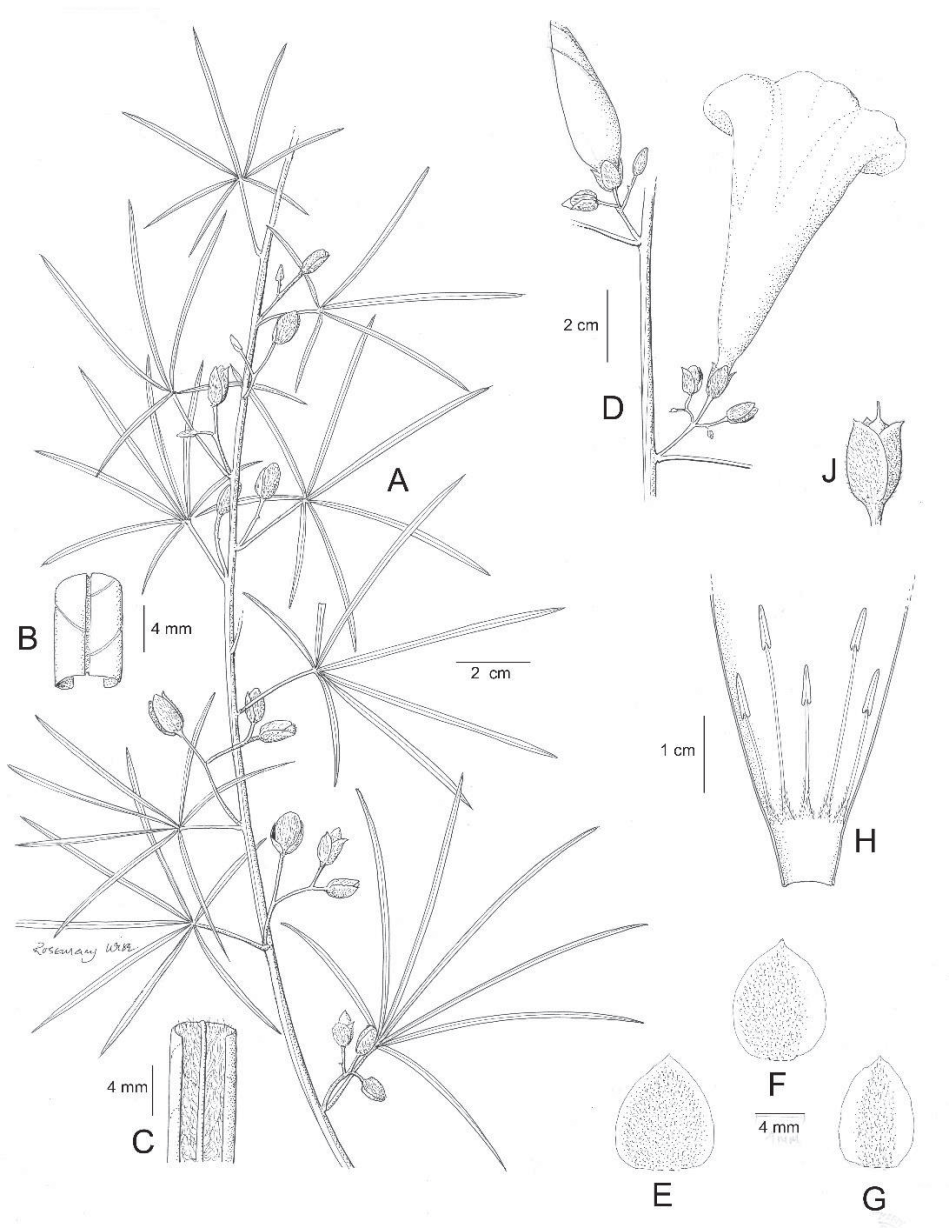
*Ipomoea revoluta*. **A** habit **B** adaxial leaf surface **C** abaxial leaf surface **D** inflorescence **E** outer sepal **F** middle sepal **G** inner sepal **H** corolla opened out to show stamens **J** calyx enclosing capsule. Drawn by Rosemary Wise from *G. Hatschbach* 23761.

#### Type.

BRAZIL. Mato Grosso do Sul, Serra de Maracaju, 17 Feb. 1970, *G. Hatschbach* 23761 (holotype MBM, isotypes CTES, F, MICH, S).

#### Description.

Slender twining liana of unknown height; stem woody, c. 2–3 mm thick, pale brown, shortly pubescent. Leaves petiolate, digitately divided into 5–7 free leaflets; leaflets 5–9 × 0.15–0.4 cm, linear, attenuate to a mucronate apex, basally tapered, margin revolute; adaxially glabrous, midvein strongly impressed; abaxially white-tomentose, the midvein prominent, nearly glabrous; petioles 8–13 mm, thinly pubescent. Inflorescence of 1–3-flowered axillary cymes; peduncles 7–9 mm, very thinly pubescent with scattered hairs; bracteoles c. 1 mm long, scale-like, caducous; pedicels 8–10 mm long, very thinly pubescent with scattered hairs; sepals subequal, 8–10 × 6–7 mm, ovate to elliptic, acute to shortly mucronate, sericeous with narrow, scarious, glabrous margins, inner sepals white-sericeous with wider scarious margins; corolla 5–6 cm long, pink, sericeous in bud, funnel shaped from a short basal cylindrical tube, limb c. 2 cm diameter, lobes rounded; stamens unequal, filaments pilose at base only, the two longer c. 20 mm, the shorter c. 12 mm; anthers included; ovary bilocular, glabrous; style c. 20 mm, stigma bi-globose. Capsule ovoid, apiculate, c. 10 mm long (immature), glabrous, ±enclosed by the sepals.

#### Distribution.

BRAZIL. Apparently endemic to the Serra de Maracaju in Mato Grosso do Sul, where it grows on arenite outcrops. Figure 9

#### Additional collection seen.

Mato Grosso do Sul, *G.M. Hatschbach & J.M. Silva* 60724 (MBM).

#### Conservation status.

The two collections were made at different dates from the near vicinity of each other. Field notes do not indicate the plant’s frequency and in the absence of other collections or any information about threats to its habitat, it can only be classified as Data Deficient (DD) within IUCN guidelines. It would be treated as a “black star” species within the classification of Hawthorne and Marshall (2016), but again this must be considered a provisional classification as no systematic search has been made for the species at the type locality or in other suitable habitats, although it must be presumed to be rare.

#### Etymology.

The epithet *revoluta* refers to the revolute margins of the leaflets.

#### Note.

We have not been able to sequence material of this species but it is almost certainly related to *I. malvaeoides* and its allies, which are part of a large clade of around 70 species almost restricted to South America. Species in the clade are characterised morphologically by the pubescent exterior of the corolla and the subequal, sericeous or pubescent, ovate herbaceous sepals. The linear leaflets of *I. revoluta* recall those of the unrelated *I. subrevoluta* Choisy, with which it has been wrongly identified in many herbaria. It is easily distinguished from that species by the sericeous exterior of the corolla and the larger, abaxially pubescent sepals.

### Ipomoea scopulina

Taxon classificationPlantaeSolanalesConvolvulaceae

J.R.I.Wood & Scotland sp. nov.

urn:lsid:ipni.org:names:77166185-1

Figure 15

#### Diagnosis.

Amongst Brazilian species of *Ipomoea*, *I. scopulina* resembles only *I. longistaminea* O’Donell and *I. ana-mariae* L.V.Vasconcelos & Sim.-Bianch in having a cylindrical suburceolate corolla but is distinguished from both of these by the broadly lanceolate subacute (not oblong-elliptic, coriaceous, somewhat concave) sepals.

#### Type.

BRAZIL. Espirito Santo, Pancas, Pedra da Colina, 19°13'51"S 40°52'35"W, 700 m, *D.P. Saraiva, J. Silva, K.V. Hmeljeviski & R.C. Forzza* 47 (holotype RB 00591205).

#### Description.

Liana of unknown height; stems woody, pale grey, glabrous. Leaves petiolate, 4–7 × 3–5 cm, ovate, shortly acuminate, base cordate with rounded auricles, margin undulate, adaxially glabrous, abaxially paler, somewhat reticulate, the main veins obscurely puberulent; petioles 1.5–2.5 cm, glabrous or obscurely puberulent upwards. Inflorescence borne on woody branchlets, the axillary cymes subracemose in form, apparently arising after the leaves have fallen; peduncles 6–7 mm long, somewhat woody, glabrous apart from a few scattered hairs; bracteoles deltoid, c. 1 mm long, glabrous, caducous; secondary peduncles 2–7 mm long; pedicels 6–10 mm long, glabrous; sepals slightly unequal, outer 6–7 × 3–3.5 mm, broadly lanceolate, subacute, glabrous, margin scarious, inner similar but obtuse and with broader scarious margins; corolla suburceolate, glabrous, reported to be “white”, 3.5–4 cm long, tube subcylindrical, c. 4 mm wide at base, widened to 10 mm in the middle, constricted upwards, c. 6 mm wide at mouth, lobes broadly ovate, c. 2 × 3.5 mm; filaments 1–1.5 cm long, glabrous except for pilose base, anthers linear, c. 2.5 mm, included; ovary presumably bilocular, glabrous, style c. 2.2 cm, stigma bi-globose. Capsule and seeds not seen.

**Figure 15.**
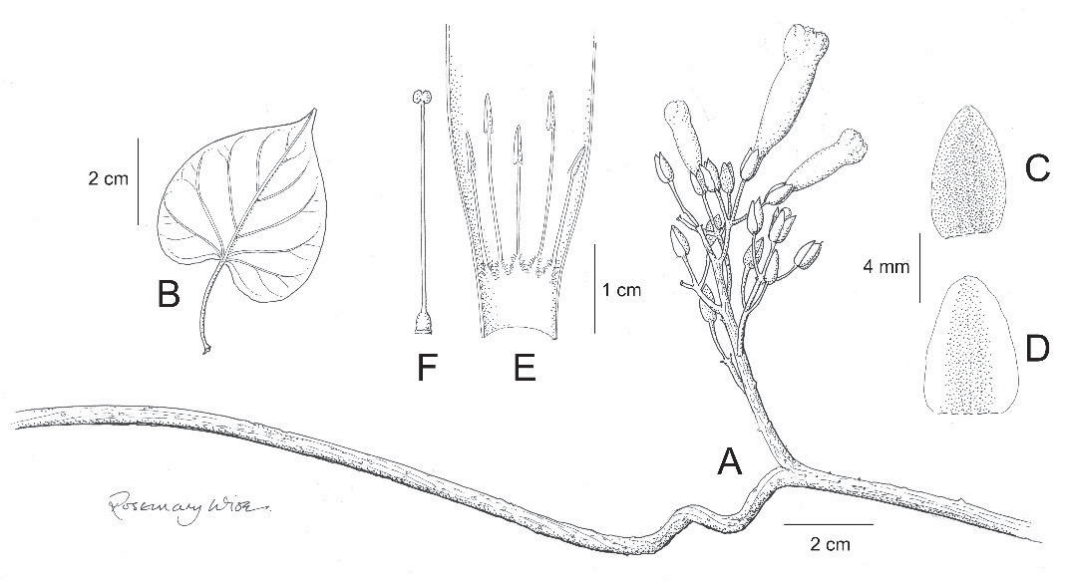
*Ipomoea scopulina*. **A** habit **B** leaf **C** outer sepal **D** inner sepal **E** corolla opened out to show stamens **F** ovary and style. Drawn by Rosemary Wise from *D.P. Saraiva et al.* 47

#### Distribution and habitat.

BRAZIL. Endemic to Espírito Santo state and only known from the type collection. It was found growing in deciduous forest amongst rock outcrops on the Pedra de Colina granite “sugarloaf” inselberg which is a feature of the country around Pancas. Like *I. fasciculata* described elsewhere in this paper, this is one of a number of *Ipomoea* species described from Bolivia and Brazil from granite inselbergs. It is to be hoped that *I. scopulina* will be found elsewhere in the same region or in similar habitats, perhaps at a considerable distance from the type locality. Figure 9

#### Conservation status.

In the absence of other collections or any information about its frequency the status of this species can only be classified as Data Deficient (DD) within IUCN (2012) guidelines. It would be treated as a “black star” species within the classification of Hawthorne and Marshall (2016), but again this must be considered as a provisional classification as no systematic search has been made for the species at the type locality or in other similar habitats in the area.

#### Etymology.

The epithet *scopulina* refers to the cliff-like sides of the granite mountain on which this species was found.

#### Notes.

The relationships of this species are not obvious but species of *Ipomoea* with tubular suburceolate corollas are generally rare in the genus and especially so in Brazil. The only two comparable Brazilian species are *I. longistaminea* and *I. ana-mariae*. Both have oblong-elliptic, coriaceous, somewhat concave sepals very different from the broadly lanceolate subacute sepals of *I. scopulina*, almost certainly indicating they belong to different clades. Additionally, *I. longistaminea* differs in its strongly exserted stamens, truncate-based, tomentellous leaves while *I. ana-mariae* differs in its oblong-elliptic cuneate-based (not ovate, cordate) leaves.

Like *I. longistaminea*, *I. scopulina* appears to be partially deciduous at anthesis. This may be significant as several species with a subcylindrical corolla from Cuba (*I. praecox* Wright) and Mexico (*I. tehuantepecensis* L. Torres, R. Torres, M.P. Ramírez & J.A. McDonald, *I. concolora* Matuda) are often leafless at anthesis.

### Ipomoea uninervis

Taxon classificationPlantaeSolanalesConvolvulaceae

J.R.I.Wood & Scotland sp. nov.

urn:lsid:ipni.org:names:77166186-1

Figure 16

#### Diagnosis.

*Ipomoea uninervis* can be confused with *I. aprica* House but differs in the grey-tomentellous, oblong outer sepals 7.5–8 mm long (these are green-tomentose, broadly ovate to suborbicular and 5–6 mm long in *I. aprica*) and the elongate inflorescence with deciduous bracts so appearing naked (not leafy with persistent bracts). It is also close to *I. oblongifolia* (Hassl.) O’Donell but differs in the 1-veined leaves (not with 3–5 prominent longitudinal veins) and oblong, not elliptic bracts, and relatively long inflorescence

#### Type.

BRAZIL. Distrito Federal, próximo ao posto Colorado, Chácara FTRC, Centro Oeste, 15°41'S, 47°52'W, 6 Feb. 1999, *C. Proença, R.S. Oliveira, C.M. Clemente, J.F. Ribeiro* 2074 (holotype UB8208-2, isotype E00202955).

#### Description.

Perennial undershrub; stems erect, to 1.2 m, sparingly branched, grey-puberulent to subsericeous. Leaves subsessile, 4–12 × 0.1–0.5 cm, linear to narrowly oblong, obtuse, shortly mucronate, both surfaces grey-puberulent to subsericeous, abaxaially paler with a single prominent longitudinal vein; petioles 0–3 mm, tomentellous. Inflorescence of few-flowered cymes from the upper leaf-axils, forming a terminal, usually elongate inflorescence up to 15 cm in length; bracts formed of reduced leaves, caducous so inflorescence appearing naked; peduncles 1–4 mm, grey-tomentellous; bracteoles 1.5 mm, linear, tomentellous, caducous; pedicels 3–7 mm, grey-tomentellous; sepals subequal, 7.5–8 × 3–4 mm, broadly oblong, obtuse to rounded, grey-tomentose, the inner with broad glabrous, scarious margins; corolla 4.5 cm long, pink, pubescent, funnel-shaped; limb c. 4 cm diameter; filaments unequal, shorter 7–10 mm, longer 12–20 mm, glabrous except for basal hairs; anthers 3.5 mm, narrow; ovary bilocular, conical, glabrous; style 15–22 mm, glabrous; stigma bilobed. Capsule and seeds not seen.

**Figure 16.**
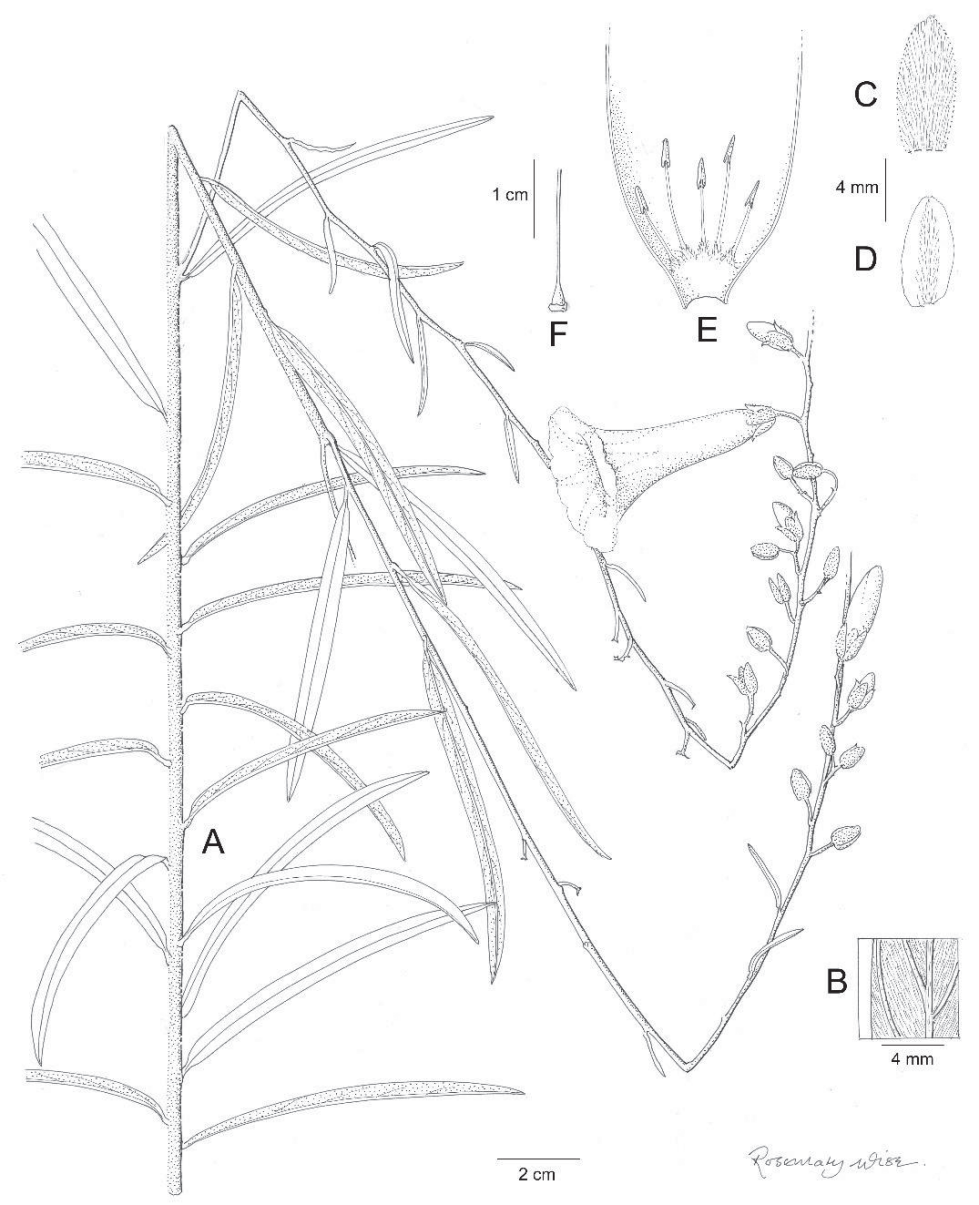
*Ipomoea uninervis*. **A** habit **B** abaxial leaf **C** outer sepal **D** inner sepal **E** corolla opened out to show stamens **F** ovary and base of style. Drawn by Rosemary Wise from *C. Proença et al.* 2074.

#### Distribution and habitat.

BRAZIL. Endemic to the Distrito Federal and Goiás State, where it appears to be a rare species of cerrado. Figure 5.

#### Additional collection seen.

BRAZIL. Goiás: Cristalina, 5 km along estrada para Paracatu, 16°46'S, 47°37'W, 1050 m, *J.R. Pirani et al.* 1560 (SPF60276, K000944736).

#### Conservation status.

This species has been found in two quite separate locations and so might be expected elsewhere. However, field notes give no information about its frequency so it should be treated as Data Deficient (DD) within IUCN guidelines until the populations of this species can be carefully evaluated.

#### Etymology.

The epithet *univervis* refers to the 1-veined leaves, the principal character separating it from *I. oblongifolia*.

#### Note.

Molecular studies using *ITS* (unpublished data) place this species in a large well-supported clade of around 70 species almost restricted to South America, which is characterised morphologically by the pubescent exterior of the corolla and the subequal, pubescent, ovate herbaceous sepals. The caducous bracts of *I. uninervis* result in a near naked inflorescence rather different from that of *I. aprica* with which it is most likely to be confused. It is probably closest to the Paraguay endemic *I. oblongifolia* from Amambay, but differs most significantly in the 1-veined leaves. *I. oblongifolia* has prominently 3–5-veined leaves.

### Ipomoea veadeirosii

Taxon classificationPlantaeSolanalesConvolvulaceae

J.R.I.Wood & Scotland sp. nov.

urn:lsid:ipni.org:names:77166187-1

Figure 17

#### Diagnosis.

*Ipomoea veadeirosii* is a densely tomentose woody liana that appears closest to *I. calyptrata* Dammer because of the persistent bracteoles which are appressed to the calyx with the pedicel supressed. It differs most obviously in the glabrous corolla, near glabrous sepals and the roughly tomentose indumentum of the leaves and stem, which differs from the white tomentellous indumentum of the stem, leaves, bracteoles, sepals and corolla exterior of *I. calyptrata*.

**Figure 17.**
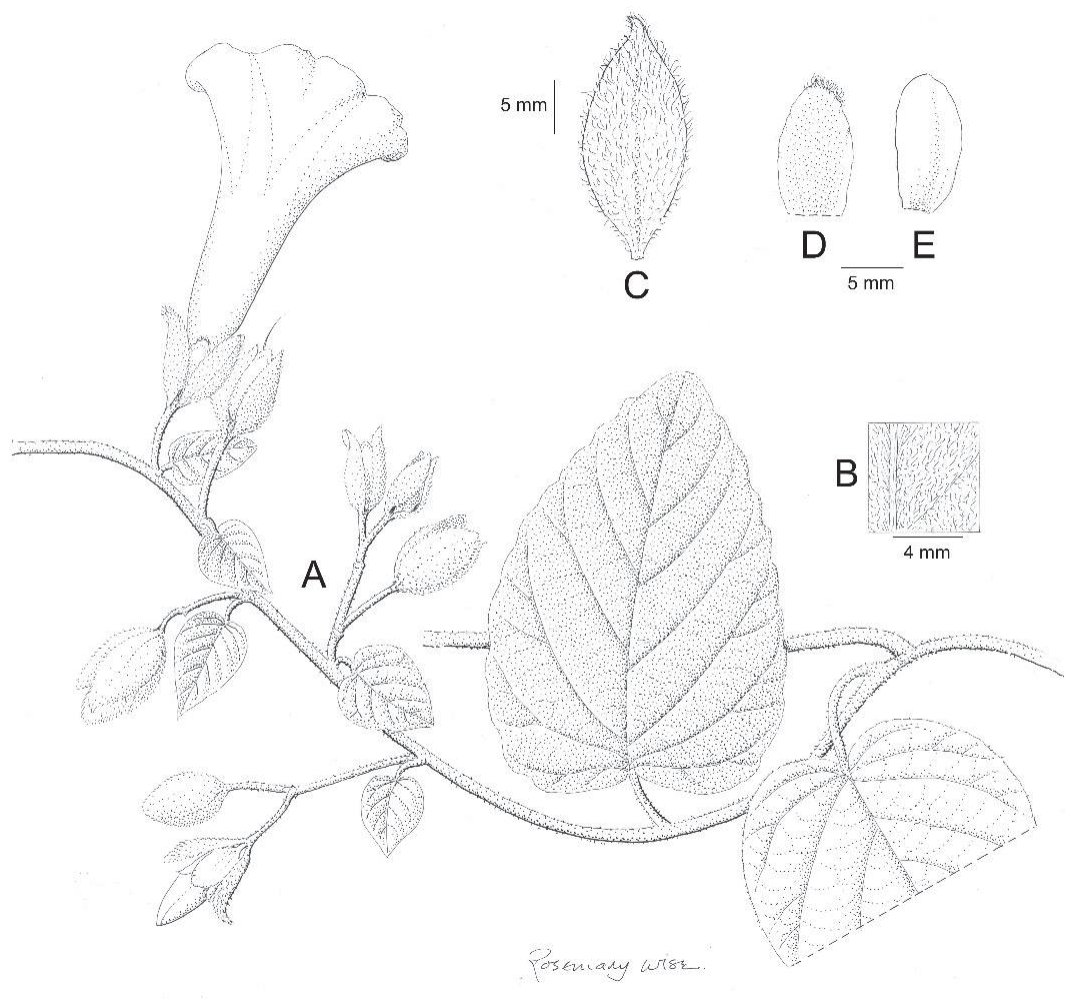
*Ipomoea veadeirosii*. **A** habit **B** abaxial leaf surface **C** bracteole **D** outer sepal **E** inner sepal. Drawn by Rosemary Wise: **A–B** from *H.S. Irwin et al.* 33148, **C–E** from *W.R. Anderson et al.* 6691.

#### Type.

BRAZIL. Goiás, Chapada dos Veadeiros, 42 km N. of Alto do Paraíso, *H.S. Irwin, R.M. Harley & G.L. Smith* 33148 (holotype FTG114415, isotype ?NY, n.v.).

#### Description.

Twining liana to c. 3 m; stem stout, somewhat woody, densely tomentose. Leaves petiolate, 5–11 × 4–9 cm, ovate, shallowly cordate to subtruncate with rounded auricles, margin undulate, apex obtuse and shortly mucronate, the mucro rather stout, adaxially yellow-green, tomentose, glabrescent when old, abaxially grey-tomentose, the veins highlighted; petioles 0.5–4 cm, tomentose. Inflorescence of flowers borne on axillary bracteate branchlets; bracts 2–2.5 × 1–1.7 cm, ovate, tomentose; cymes 1–2-flowered; peduncles 1–6 cm, tomentose; secondary peduncles pedicel-like, 0.8–1.7 cm, pubescent, more slender than primary peduncles; bracteoles 2–2.3 × 0.8–1.4 cm, narrowly elliptic, obtuse, somewhat boat-shaped, tomentose, persistent and ± clasping the calyx; pedicels 1–4 mm, glabrous; sepals subequal, 11–13 × 5–7 mm, elliptic, obtuse to rounded, outer glabrous, margins scarious; corolla 6–7 cm, narrowly funnel-shaped, glabrous, deep pink.

#### Distribution and habitat.

BRAZIL. Endemic to rocky cerrado (campo rupestre?) at 1250–1700 m in the Chapada dos Veadeiros National Park. Figure 9.

#### Additional collection seen.

Goiás: Chapada dos Veadeiros, 25 km N of Alto Paraíso, 1700 m, *W.R. Anderson et al.* 6691 (FTG114414, ?NY, n.v.)

#### Conservation status.

The two collections were made on different dates from two nearby locations. Field notes do not indicate the plant’s frequency and in the absence of other collections or any information about threats to its habitat, it can only be classified as Data Deficient (DD) within IUCN (2012) guidelines. It would be treated as a “black star” species within the classification of Hawthorne and Marshall (2016), but again this must be considered a provisional classification as no systematic search has been made for the species at the type locality or in other suitable habitats, although it must be presumed to be rare. Both recorded locations lie within the Chapada dos Veadeiros National Park and enjoy legal protection.

#### Etymology.

This species is named after the Chapada dos Veadeiros National Park in Goiás State. It is one of the biologically richest and most important protected areas of the cerrado biome and four species described in this paper are endemic to this National Park.

#### Note.

Although we have not been able to sequence this species, *I. veadeirosii* appears to belong to a small clade consisting of *I. descolei* O’Donell, *I. marcellia* Meisn. and *I. calyptrata*. All these species are somewhat woody and liana-like and share a densely tomentose indumentum. The inflorescence structure, with a tendency for the inflorescence to develop on foliose branchlets, is found in a number of woody species, notably in the *Arborescens* clade. *Ipomoea veadeirosii* appears closest to *I. calyptrata* because of the persistent bracteoles which are appressed to the calyx with the pedicel supressed but is readily distinguished from all these species by the glabrous exterior of the corolla.

### Ipomoea velutinifolia

Taxon classificationPlantaeSolanalesConvolvulaceae

J.R.I.Wood & Scotland

Figure 18

#### Diagnosis.

*Ipomoea velutinifolia* is apparently related to *I. sericosepala* J.R.I. Wood & Scotland because of the compound inflorescence and the distribution of the indumentum on the corolla and almost all vegetative parts but differs in the distinctive velvety grey indumentum and in the shape and size of the sepals which are subequal, ovate to suborbicular, 7–8 × 6–8 mm, not unequal, elliptic-obovate, the outer oblong 8–10 × 3–4 mm and the inner 11–14 × 6 mm. The pubescent ovary is a very unusual character in *Ipomoea*.

#### Type.

BRAZIL. Maranhão: Mun. Grajau, 4 km W of Mondelandia on path to Rio Grajaú, 23 April 1983, *E.L. Taylor, C.S. Rosario & J.B.F. Silva* 1326 (holotype MG114153, isotype ARIZ421888).

#### Description.

Perennial climber, stems relatively stout, silky-velutinous. Leaves petiolate, 5–9 × 4–8 cm, ovate, apex acute, mucronate, base very broadly cuneate to subtruncate with rounded auricles, margin undulate, adaxially softly and densely pubescent, abaxially velvety-grey; petioles 2.5–4.5 cm, velvety-grey. Inflorescence of compound axillary cymes, these often racemose in form and sometimes distinctly leafy; peduncles 2.5–5 cm, velvety-grey, often extended as a rhachis and reaching 15 cm; secondary peduncles 0.5–2 cm, velvety-grey; bracteoles caducous, not seen; pedicels 10–12 mm, puberulent; sepals subequal, 7–8 × 6–8 mm, outer ovate, obtuse, inner suborbicular, rounded, abaxially velvety-grey, adaxially glabrous; corolla 4.5–6 cm long, sericeous, funnel-shaped, exterior white, interior pale pink; stamens unequal, included, longer c. 2.5 cm, shorter c. 1.2 cm, anthers linear 5 mm; ovary pubescent; stigma not seen. Capsule and seeds unknown.

**Figure 18.**
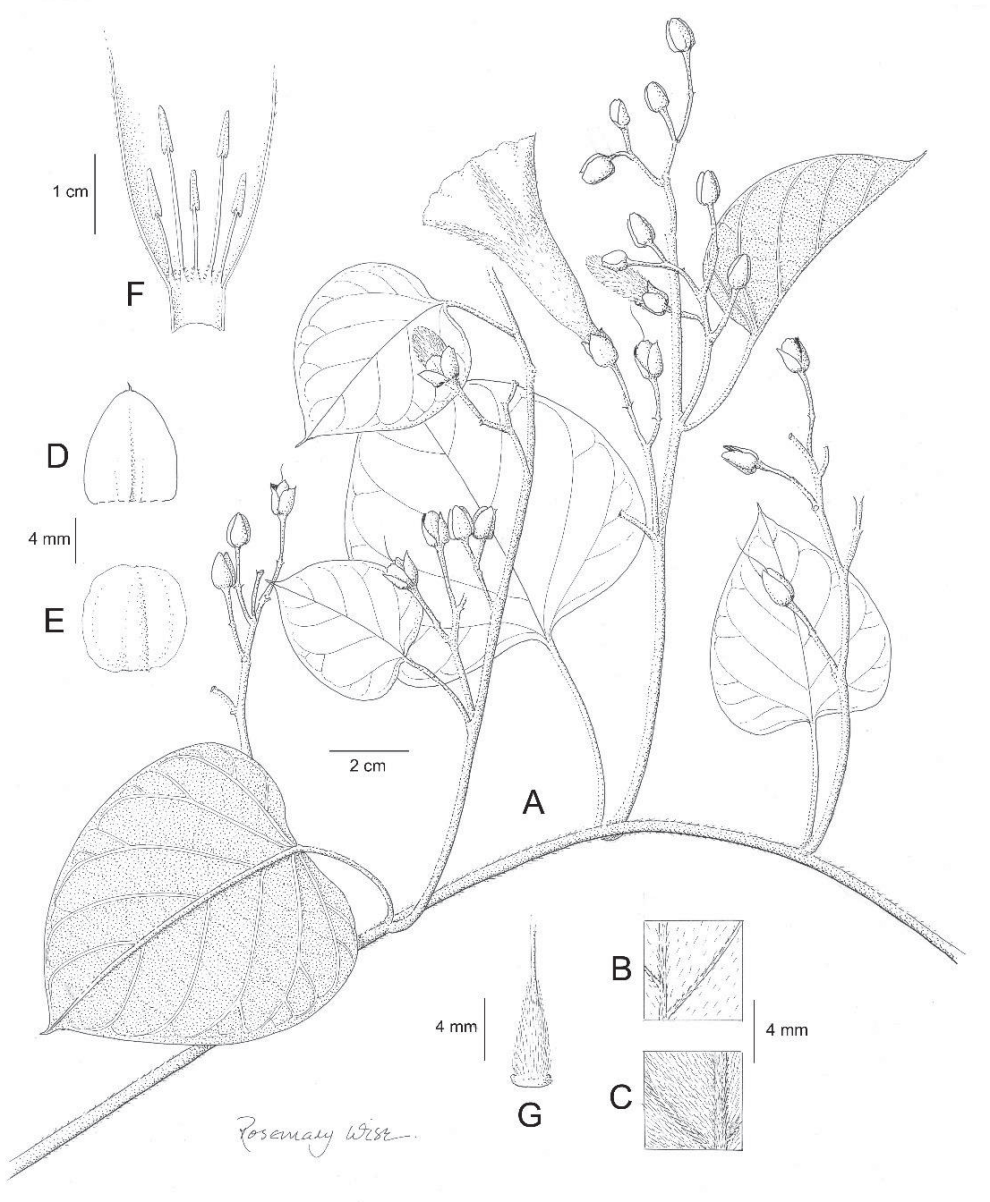
*Ipomoea velutinifolia*. **A** habit **B** adaxial leaf surface **C** abaxial leaf surface **D** outer sepal **E** inner sepal **F** corolla opened out to show stamens **G** ovary. Drawn by Rosemary Wise from *E.L. Taylor, et al.* 1326.

#### Distribution and Habitat.

BRAZIL and PERU. Known from the type locality in Maranhão and a single locality at low altitudes in Pasco. Unlike most of the other species in this paper this is a forest species, found in Brazil in the only mature forest relic on a ridge and in Peru in primary forest on white sands. The forest in Brazil was reported to be relatively short with few emergent trees, an open understorey and the presence of many vines in areas where trees had fallen. The distribution (Figure 5) is very disjunct but suggests that this species might be found in other suitable habitats around the Amazon basin.

#### Additional collection.

PERU. Pasco: Oxapampa, Palcazu Dist, San Cristobal, 323 m, 29 May 2008, *R. Vásquez et al.* 34378 (MO, USM); ibid., Comunidad Nativa Buenos Aires, *R. Vásquez et al.* 37328 (MO, OXF).

#### Conservation status.

The two widely separated locations for this species combined with the relatively recent collection dates suggest this species may have been overlooked. No information is available to indicate the plant’s frequency but field notes accompanying the type collection indicate that its forest habitat is threatened. It would be treated as a “black star” species in both Brazil and Peru within the classification of Hawthorne and Marshall (2016), but within IUCN (2012) guidelines it can only be classified as Data Deficient (DD). However, this must be considered only a provisional classification until the populations can be assessed.

#### Etymology.

The epithet *velutinifolia* refers to the softly velvety indumentum of the leaves.

#### Note.

This species appears to be related to *I. sericosepala* but differs in the subequal sepals and the grey-green velutinous indumentum. It might be interpreted as being related to *I. megapotamica* Choisy or *I. sericophylla* but differs from both in the form of the inflorescence and indumentum. From all these species and most others known from South America it also differs in the densely pubescent ovary.

### Ipomoea walteri

Taxon classificationPlantaeSolanalesConvolvulaceae

J.R.I.Wood & Scotland sp. nov.

urn:lsid:ipni.org:names:77166188-1

Figure 19

#### Diagnosis.

*Ipomoea walteri* is close to *I. sericophylla* but distinct because of the long-pedunculate lax inflorescence, adaxially nearly glabrous leaves and unequal sepals, the inner noticeably shorter than the outer. Particularly distinct are the strongly cuspidate leaves with a distinct apical mucro c. 3 mm long which is only matched in a few very different species, such as *I. daturiflora* Meisn.

#### Type.

BRAZIL. Goiás: Colinas do Sul, arredores da Serra de Jipe, 500 m, *B.M.T. Walter, E. Gomes, G. Pereira-Silva & S. Pereira de Souza* 4734 (CEN58673).

#### Description.

Liana of unknown height, stems thinly pubescent; leaves petiolate, 3–5 × 3.5–5.5 cm, ovate, apex obtuse and long-cuspidate (mucro c. 3–4 mm), base cordate with rounded auricles, adaxially very sparsely pubescent to subglabrous, abaxially grey tomentose, gland-dotted; petioles 2.5–3.5 cm. Inflorescence of long-pedunculate lax axillary cymes; peduncles 7–11 cm; bracteoles caducous, not seen, secondary peduncles 0.3–2.2 cm; tertiary peduncles c. 10 mm; pedicels 4–5 mm; sepals unequal, outer 11–12 × 8–9 mm, obovate-elliptic, rounded, thinly tomentellous; inner 8–9 × 6 mm, the central part densely tomentose, margins broad, glabrous, scarious; corolla 5.5 cm long, appearing broadly tubular but not fully open, probably funnel-shaped when open, pale pink; stamens unequal, longer c. 1.5–1.8 cm, shorter c. 1–1.2 cm; anthers 3 mm, included; style 2–2.3 cm, stigma bi-globose.

**Figure 19.**
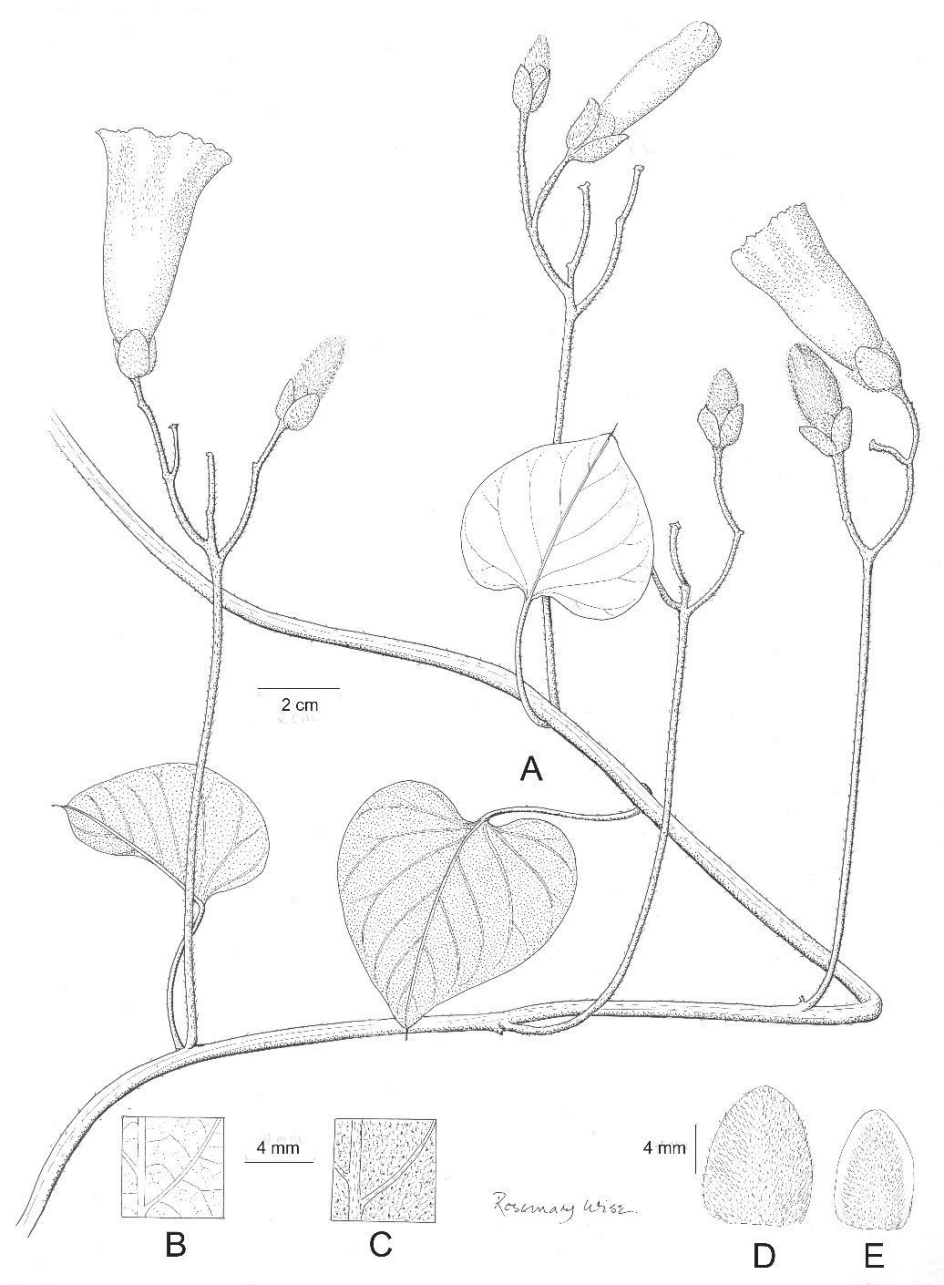
*Ipomoea walteri*. **A** habit **B** adaxial leaf surface **C** abaxial leaf surface **D** outer sepal **E** inner sepal. Drawn by Rosemary Wise from *B.M.T. Walter et al.* 4734.

#### Distribution and habitat.

BRAZIL. Goiás. Known only from the type collection. It was recorded as growing in gallery forest. Figure 9.

#### Conservation status.

Field notes give no data about the frequency of this species and in the absence of other collections or any information about threats to its habitat, it can only be classified as Data Deficient (DD) within IUCN guidelines. It would be treated as a “black star” species within the classification of Hawthorne and Marshall (2016), but again this must be considered a provisional classification as no systematic search has been made for the species at the type locality or in other suitable habitats, although it must be presumed to be rare.

#### Etymology.

This species is named after the collector Bruno Walter, who is a leading research worker for Embrapa at the Cenargen Herbarium in Brasilia and a specialist in the Flora of the Cerrado.

#### Note.

Although we have not been able to sequence this species, *I. walteri* clearly belongs to the large clade of around 70 species almost restricted to South America, which is characterised morphologically by the pubescent exterior of the corolla and the subequal, pubescent, ovate herbaceous sepals. The strongly cuspidate leaves with a distinct apical mucro are particularly distinctive.

## Supplementary Material

XML Treatment for Ipomoea attenuata

XML Treatment for Ipomoea cuscoensis

XML Treatment for Ipomoea dasycarpa

XML Treatment for Ipomoea dolichopoda

XML Treatment for Ipomoea ensiformis

XML Treatment for Ipomoea fasciculata

XML Treatment for Ipomoea graminifolia

XML Treatment for Ipomoea kraholandica

XML Treatment for Ipomoea longirostra

XML Treatment for Ipomoea revoluta

XML Treatment for Ipomoea scopulina

XML Treatment for Ipomoea uninervis

XML Treatment for Ipomoea veadeirosii

XML Treatment for Ipomoea velutinifolia

XML Treatment for Ipomoea walteri
